# Long-term clinical sequelae in severe fever with thrombocytopenia syndrome: A longitudinal cohort study

**DOI:** 10.1371/journal.pntd.0013276

**Published:** 2025-08-12

**Authors:** Ning Cui, Xin Yang, Hong-Han Ge, Xiao-Hong Yin, Yi-Mei Yuan, Chao Zhou, Xi Wang, Hai-Feng Pan, Hao Li, Xiao-Ai Zhang, Li-Qun Fang, Li-Fen Hu, Peng-Tao Bao, Wei Liu

**Affiliations:** 1 The 154th Hospital, China RongTong Medical Healthcare Group Co.Ltd, Xinyang, China; 2 State Key Laboratory of Pathogen and Biosecurity, Academy of Military Medical Science, Beijing, China; 3 School of Public Health, Shandong First Medical University and Shandong Academy of Medical Sciences, Jinan, China; 4 School of Public Health, Anhui Medical University, Hefei, China; 5 The First Affiliated Hospital of Anhui Medical University, Hefei, China; 6 The Eighth Medical Center of Chinese PLA General Hospital, Pulmonary and Critical Care Medicine Faculty of Chinese PLA General Hospital, Beijing, China; Huadong Research Institute for Medicine and Biotechniques, CHINA

## Abstract

**Background:**

Severe fever with thrombocytopenia syndrome (SFTS) is an emerging tick-borne infectious disease characterized by a high case fatality rate. Despite extensive research on acute-phase manifestations, the long-term clinical sequelae in survivors remain poorly characterized.

**Methods:**

In this prospective cohort study from 2010 to 2024, 1,197 SFTS survivors and 188 age/sex-matched febrile controls without SFTS were enrolled from the highest endemic region in China. Participants underwent face-to-face interview, serial clinical evaluations and laboratory testing at 6, 12, 18 and 24 months post-discharge, with extended follow-up for a subset (n = 294) over 11 years. Propensity score matching and multivariate logistic regression were used to determine the factors associated with long-term sequelae risk.

**Results:**

A total of 62.57% (749/1,197) of survivors developed persistent sequelae, significantly higher than controls (51.60%, 97/188; *P* < 0.05). Key manifestations included memory impairment (33.50%, 401/1,197), arthralgia (33.08%, 396/1,197), alopecia (32.25%, 386/1,197) and visual decline (31.08%, 372/1,197). Laboratory abnormalities persisted for ≥10 years in 0.33% of survivors, notably thrombocytopenia, elevated lactate dehydrogenase, and cystatin C. Compared to non-SFTS group, a significantly higher proportion of SFTS survivors had decreased white blood cell count, eosinophil percentage and mean corpuscular hemoglobin. The long-term sequelae risk exhibited distinct patterns across factors: encephalitis development was associated with significantly higher risks of memory impairment (adjusted OR = 2.39) and thrombocytopenia (adjusted OR = 3.36); corticosteroid usage during hospitalization showed increased risks of arthralgia (adjusted OR=2.17) and elevated BUN (adjusted OR=3.87); while high viral load (≥1 × 10^6^ copies/mL) exhibited significantly higher incidences of most prevalent clinical manifestations and multiple laboratory abnormalities (all **P* *< 0.05).

**Conclusion:**

SFTS survivors exhibit multisystemic sequelae, with high viral load and acute-phase neurological involvement serving as critical prognostic indicators. These findings underscore the need for long-term monitoring and targeted therapeutic strategies for SFTS.

## Author summary

Our study reveals the long-term health consequences of Severe fever with thrombocytopenia syndrome (SFTS) in survivors, demonstrating that over 60% develop persistent symptoms such as memory impairment, arthralgia, and visual decline, with some laboratory abnormalities lasting up to a decade. Key risk factors include high viral load, acute-phase encephalitis, and corticosteroid use, while antiviral treatment (e.g., favipiravir) may reduce certain sequelae. These findings highlight SFTS as not only an acute infection but also a chronic condition requiring long-term monitoring and tailored rehabilitation strategies. Our results emphasize the need for improved survivor care and cautious therapeutic approaches during acute illness to mitigate lasting health impacts.

## Introduction

Severe fever with thrombocytopenia syndrome (SFTS), caused by SFTS virus (SFTSV), taxonomically designated as *Dabie bandavirus* (DBV), genus *Bandavirus,* family *Phenuiviridae*, order *Bunyavirales* [1], is an emerging tick-borne infectious disease of significant public health concern. First identified in rural China in 2009 [2], SFTS has since been epidemiologically confirmed across multiple Asian countries, including South Korea, Japan, and Vietnam, etc [3–7]. The virus is maintained in enzootic cycles involving *Haemaphysalis longicornis* ticks, through both vertical and horizontal transmission [8]; with wild and domestic mammals serving as amplifying hosts, which develop transient viremia without overt clinical manifestations [9]. Human infection primarily occurs through bites by the ticks that carry SFTSV [10]. Additionally, human-to-human transmission of SFTS has been widely reported, often resulting from unprotected exposure to those with severe SFTS disease [11,12].

Clinical manifestations of SFTS range from mild nonspecific febrile illnesses to severe complications and even death [13–15]. The disease exhibits a rapid progression, starting with nonspecific symptoms such as fever, gastrointestinal symptoms, headache, myalgia, etc, before advancing to a hemorrhagic phase characterized by overt bleeding, disseminated intravascular coagulation, and organ failure, ultimately leading to shock. Fatal outcome usually occurs during the second week following symptom onset [16,17], with an estimated case fatality rate (CFR) ranging from 10% to 30% [13,18,19]. However, CFR reported by the China Information System for Disease Control and Prevention (CISDCP) is remarkably lower due to the exclusion of patients who leave the hospital against medical advice, despite their high risk of fatal outcomes [13]. In survivors, clinical recovery generally begins around 9–10 days post-onset, marked by the resolve of clinical symptoms/syndromes and normalization of laboratory parameters [16]. Survivors might develop robust humoral immunity against SFTSV, which that can persist for several years, providing protection against reinfection [20,21]. Until recently, there has been no substantial evidence of relapse or a biphasic course of SFTS, exception for a single case report documenting SFTSV reinfection in one female patient [22]. However, the long-term sequelae or complications that may arise in survivors remain poorly understood due to the lack of follow-up observation.

During the 2010–2024, we conducted a prospective observational study on a cohort of individuals who had survived SFTSV infection. By comparing these survivors with a control group of febrile patients without SFTSV infection, we investigated the development of clinical sequelae associated with SFTSV infection. Furthermore, clinical sequelae that might be related to severe disease, medication use during the acute phase, and complications such as encephalitis and hemorrhage during hospitalization were further explored by comparing among the SFTS survivors.

## Methods

### Ethical statement

The research protocol was approved by the Ethical Committee of Academy of Military Medical Sciences (AF/SC-08/02.72), with written informed consent obtained from each participant.

### Study population

This multicenter prospective cohort study was conducted at the 154^th^ Hospital and Shangcheng Hospital in Xinyang City, Henan Province, both of which serve as the referral centers for SFTS patients in Henan Province. Between 2010 and 2024, all laboratory-confirmed SFTS patients discharged from these centers were eligible for inclusion. Diagnosis criteria adhered to the following: (1) clinical manifestations consistent with SFTS (i.e., high fever accompanied by thrombocytopenia and leukopenia, gastrointestinal symptoms) and either (2) a positive polymerase chain reaction result for SFTSV or (3) a four-fold increase in antibody levels between paired serum samples. Additionally, the control group were selected from the same hospitals during the same years. These individuals were hospitalized due to febrile illnesses that resembled SFTS but tested negative for SFTSV via PCR and did not develop SFTSV-specific IgM antibodies.

All enrolled participants, including both former confirmed SFTS cases and control participants, were alive at the time of discharge. After obtaining informed consent from each participant, epidemiological data and past clinical information were retrieved from medical records and through patient interviews.

### Follow-up study

Participants underwent follow-up evaluation at 6, 12, 18 and 24 months post- disease onset, with optimal extended evaluations for a subset (n = 294) over 11-year longitudinal period. Trained physicians administered standardized face-to-face interviews using a validated symptom inventory questionnaire to systematically document newly occurring, persistent, and progressive clinical manifestations attributable to SFTSV infection. Venous blood samples were collected at each visit and analyzed using the following protocols. Hematologic parameters that measure platelet (PLT) count, leukocyte differentials, and hemoglobin levels; hepatic function panel that measure alanine aminotransferase (ALT), aspartate aminotransferase (AST), and lactate dehydrogenase (LDH); renal function panel that measure uric acid (UA), blood urea nitrogen (BUN), and cystatin C (CYSC) levels. Exclusion criteria include: (1) Aged <18 years or pregnancy/lactation; (2) Loss to follow up; (3) Incomplete laboratory tests during follow-up; (4) Voluntary withdrawal from the study post-discharge.

### Grouping strategy and severity stratification

**Fig 1:**
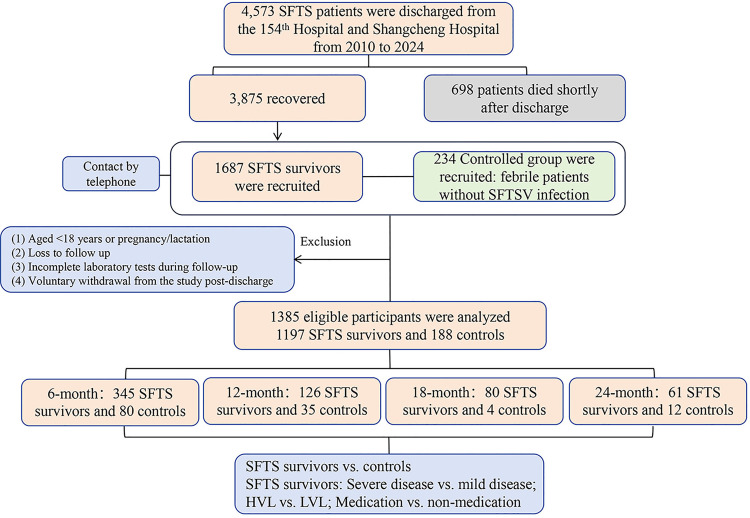
The flowchart of recruiting and grouping patients. Severe disease was defined as the presence of one or more of the following complications: encephalitis, hemorrhagic manifestations, or high viral load; mild disease was defined as the absence of these complications. Follow-up durations were categorized as follows: 6-month (within 6 months or between 6 and 8 months after disease onset), 12-month (between 8 and 12 months), 18-month (between 12 and 18 months), and 24-month (between 18 and 24 months). The LVL group referred to patients with a viral load ≤ 1 × 10^6^ copies/mL on admission; the HVL group referred to patients with a viral load **≥** 1 × 10^6^ copies/mL on admission. The medication and non-medication groups were defined based on whether patients received treatments such as favipiravir, ribavirin, and corticosteroids during the acute phase.

To address attrition bias inherent in longitudinal studies, participants were stratified into four cohorts based on follow-up completion intervals. The 6-month group: Evaluated within 0–8 months post-symptom onset. The 12-month group: Assessed at 8–12 months. The 18-month group: Followed at 12–18 months. The 24-month group: Tracked at 18–24 months. Severity stratification was applied based on encephalitis and hemorrhagic manifestation, measurement of viral load according to our previous definition on severe SFTS [19]. Encephalitis was defined as the presence of consciousness or meningeal irritation sign [15]. Hemorrhagic manifestations were defined as the occurrence of any of the following: bloody stool, gingival bleeding, hemoptysis, hematemesis, epistaxis, oral bleeding, gross hematuria, ocular bleeding, etc. Patients were categorized into low viral load group (LVL) and high viral load group (HVL) based on the median viral load at admission for all hospitalized SFTS patients (1 × 10^6^ copies/mL), with LVL defined as ≤ 1 × 10^6^ copies/mL and HVL as > 1 × 10^6^ copies/mL. Additionally, patients were grouped based on whether they received treatments such as favipiravir, ribavirin, and corticosteroids during hospitalization. The flowcharts illustrating the patient grouping process are presented in Fig 1.

### Statistical analysis

Continuous variables were reported as medians with interquartile ranges (IQR), and comparisons between groups were performed using the Wilcoxon rank-sum test. Categorical variables were summarized as frequencies and percentages, with chi-square tests used for between-group comparisons. Propensity score matching (PSM) was performed at a 1:1 ratio to balance baseline characteristics, including age, sex, and underlying diseases, between patients with and without encephalitis/hemorrhagic symptoms, as well as between the medication and non-medication groups during hospitalization. A nearest-neighbor matching algorithm with a caliper width of 0.2 standard deviations of the logit of the propensity score was used to ensure balanced comparability of baseline features. Multivariate logistic regression analysis was also conducted, adjusting for potential confounders such as age, sex, time from disease onset to treatment, and comorbidities, to estimate the association between sequelae and patient groups or subgroups. The analysis generated odds ratios (ORs) with 95% confidence intervals (CIs). All statistical analyses were performed using R version 4.1.2 (R Foundation for Statistical Computing, Vienna, Austria), with a two-sided *P* value less than 0.05 considered statistically significant.

## Results

### The enrolled participants

Between June 2010 and November 2024, a total of 4573 patients diagnosed with SFTS were discharged from 154^th^ Hospital and Shangcheng Hospital. Notably, 698 patients (15.26%) died from SFTS-related complications after discontinuing therapy and leaving the hospital, with a median time from hospital discharge to death of three days. Of the remaining survivors (n = 3875), 1687 adult patients were successfully contacted for potential study participation, from whom 1197 SFTS survivors and 188 control individuals underwent face-to-face interviews, physical examinations, and laboratory tests. Demographic and clinical characteristics during the follow-up period are summarized in Table 1. The median age of SFTS survivors at follow-up was 65 years (IQR: 56–72), with 440 males (36.76%) and 757 females (63.24%). Compared to the non-SFTS group, SFTS survivors were older, had a higher proportion of females, and a higher prevalence of comorbidities. Comparisons between recruited and non-recruited participants revealed comparable age, gender, presence of main comorbidities, and disease severity during hospitalization (S1 Table).

**Table 1:** Summary of participant demographics and follow-up information.

Characteristics	Total Population	Uninfected Controls	SFTS Survivors	*P *value
(N = 1385)	(N = 188)	(N = 1197)
Gender, Female: n (%)	863 (62.31%)	106 (56.38%)	757 (63.24%)	0.085
Age at Onset, years	61 (52, 68)	57 (49, 64)	62 (52, 69)	<0.001
Age at Follow-up, years	64 (55, 72)	59 (51, 68)	65 (56, 72)	<0.001
Time Interval (Median, Interquartile Range)				
Days from Onset to Hospital Admission	4 (3, 6)	3 (1, 5)	5 (3, 6)	<0.001
Days from Hospital Admission to Discharge	8 (6, 10)	6 (4, 8)	9 (7, 11)	<0.001
Months from Onset to Follow-up	20 (5, 68)	8 (4, 51)	22 (6, 74)	<0.001
Comorbidities, n (%)				
Any Comorbidity	548 (39.57%)	58 (30.85%)	490 (40.94%)	0.011
Hypertension	181 (13.07%)	14 (7.45%)	167 (13.95%)	0.019
Diabetes	98 (7.08%)	14 (7.45%)	84 (7.02%)	0.957
Chronic Obstructive Pulmonary Disease	80 (5.78%)	14 (7.45%)	66 (5.51%)	0.656
Hepatitis	107 (7.73%)	10 (5.32%)	97 (8.10%)	0.235
Cardiovascular Disease	74 (5.34%)	8 (4.26%)	66 (5.51%)	0.590
Follow-up Symptoms, n (%)				
Memory Impairment	444 (32.06%)	43 (22.87%)	401 (33.50%)	0.005
Alopecia	433 (31.26%)	47 (25.00%)	386 (32.25%)	0.066
Arthralgia	439 (31.70%)	43 (22.87%)	396 (33.08%)	0.008
Visual Impairment	414 (29.89%)	42 (22.34%)	372 (31.08%)	0.019
Fever	30 (2.17%)	4 (2.13%)	26 (2.17%)	0.623
Dizziness	24 (1.73%)	1 (0.53%)	23 (1.92%)	0.248
Headache	31 (2.24%)	3 (1.60%)	28 (2.34%)	0.610
Fatigue	90 (6.50%)	18 (9.57%)	72 (6.02%)	0.165
Hearing Loss	13 (0.94%)	2 (1.06%)	11 (0.92%)	1.000
Myalgia	38 (2.74%)	5 (2.66%)	33 (2.76%)	1.000
Any Symptom	846 (61.08%)	97 (51.60%)	749 (62.57%)	0.005
Follow-up Abnormal Laboratory Findings, n (%)				
Blood Routine Examination				
WBC↓	172 (12.42%)	22 (11.70%)	150 (12.53%)	0.749
PLT↓	136 (9.82%)	14 (7.45%)	122 (10.19%)	0.254
NEUT%↓	211 (15.23%)	26 (13.83%)	185 (15.46%)	0.549
LYM%↓	136 (9.82%)	15 (7.98%)	121 (10.11%)	0.379
MONO%↓	49 (3.54%)	3 (1.60%)	46 (3.84%)	0.165
EOS%↓	120 (8.66%)	7 (3.72%)	113 (9.44%)	0.011
MCH↓	75 (5.42%)	3 (1.60%)	72 (6.02%)	0.018
RDW↑	19 (1.37%)	2 (1.06%)	17 (1.42%)	0.914
Liver Function Tests				
ALT↑	92 (6.64%)	17 (9.04%)	75 (6.27%)	0.206
AST↑	86 (6.21%)	14 (7.45%)	72 (6.02%)	0.528
GGT↑	119 (8.59%)	16 (8.51%)	103 (8.60%)	1.000
LDH↑	256 (18.48%)	24 (12.77%)	232 (19.38%)	0.038
TBA↑	76 (5.49%)	14 (7.45%)	62 (5.18%)	0.243
Renal Function Tests				
BUN↑	89 (6.43%)	16 (8.51%)	73 (6.10%)	0.651
UA↑	154 (11.12%)	29 (15.43%)	125 (10.44%)	0.421
CYSC↑	364 (26.28%)	47 (25.00%)	317 (26.48%)	0.382

Note: Data are n (%) unless otherwise specified. Categorical variables were compared between groups using χ2 tests. Continuous variables were presented as medians with interquartile ranges (IQR) and were compared using the Mann-Whitney U test. *P* values less than 0.05 were considered statistically significant. The symbols ‘↓’ and ‘↑’ indicate laboratory values below and above the normal range, respectively.

Abbreviations: ALT, alanine aminotransferase; AST, aspartate aminotransferase; BUN, blood urea nitrogen; CYSC, cystatin C; EOS%, eosinophil percentage; GGT, gamma-glutamyltransferase; LDH, lactate dehydrogenase; LYM%, lymphocyte percentage; MCH, mean corpuscular hemoglobin; MONO%, monocyte percentage; NEUT%, neutrophil percentage; PLT, platelet count; RDW, red cell distribution width; TBA, total bile acid; UA, uric acid; WBC, white blood cell count.

The median duration from symptom onset to the follow-up visit was 22 months (IQR: 6–74). At the 6-month follow-up, 425 participants were successfully evaluated, including 345 SFTS-survivors (101 with severe disease, 244 with mild disease) and 80 controls. At the 12-month follow-up, 161 participants were assessed, including 126 survivors (95 with severe disease, 31 with mild disease) and 35 controls (Table 2). At the 18-month follow-up, 84 participants were successfully evaluated, including 80 SFTS-survivors (25 with severe disease, 55 with mild disease) and 4 controls. At the 24-month follow-up, 73 participants were assessed, including 61 survivors (18 with severe disease, 43 with mild disease) and 12 controls (Fig 1).

**Table 2:** Prevalence of clinical symptoms and laboratory findings on physical examination.

Characteristics	Uninfected Controls vs. SFTS Survivors				Mild Cases vs. Severe Cases			
6-month		12-month		6-month		12-month	
Total Number per Group	80 vs. 345		35 vs. 126		244 vs. 101		95 vs. 31	
Clinical Symptoms		*P* value		*P* value		*P* value		*P* value
Alopecia	11 (13.75%) vs. 115 (33.33%)	<0.001	3 (8.57%) vs. 37 (29.37%)	0.022	80 (32.79%) vs. 35 (34.65%)	0.834	29 (30.53%) vs. 8 (25.81%)	0.784
Memory Impairment	19 (23.75%) vs. 92 (26.67%)	0.694	4 (11.43%) vs. 40 (31.75%)	0.033	68 (27.87%) vs. 24 (23.76%)	0.515	29 (30.53%) vs. 11 (35.48%)	0.769
Arthralgia	8 (10.00%) vs. 85 (24.64%)	0.007	6 (17.14%) vs. 47 (37.30%)	0.041	62 (25.41%) vs. 23 (22.77%)	0.704	33 (34.74%) vs. 14 (45.16%)	0.408
Visual Impairment	9 (11.25%) vs. 83 (24.06%)	0.019	10 (28.57%) vs. 38 (30.16%)	0.981	61 (25.00%) vs. 22 (21.78%)	0.617	28 (29.47%) vs. 10 (32.26%)	0.946
Abnormal Laboratory Findings								
Blood Routine Examination								
WBC↓	4 (5.00%) vs. 54 (15.65%)	0.023	4 (11.43%) vs. 14 (11.11%)	1.000	34 (13.93%) vs. 20 (19.80%)	0.229	13 (13.68%) vs. 1 (3.23%)	0.217
PLT↓	7 (8.75%) vs. 33 (9.57%)	0.898	4 (11.43%) vs. 15 (11.90%)	1.000	21 (8.61%) vs. 12 (11.88%)	0.459	12 (12.63%) vs. 3 (9.68%)	0.959
NEUT%↓	11 (13.75%) vs. 59 (17.10%)	0.475	4 (11.43%) vs. 23 (18.25%)	0.335	42 (17.21%) vs. 17 (16.83%)	1.000	16 (16.84%) vs. 7 (22.58%)	0.577
LYM%↓	7 (8.75%) vs. 31 (8.99%)	1.000	3 (8.57%) vs. 13 (10.32%)	0.850	20 (8.20%) vs. 11 (10.89%)	0.556	9 (9.47%) vs. 4 (12.90%)	0.782
MONO%↓	3 (3.75%) vs. 15 (4.35%)	0.994	0 (0.00%) vs. 4 (3.17%)	0.591	10 (4.10%) vs. 5 (4.95%)	0.950	3 (3.16%) vs. 1 (3.23%)	1.000
EOS%↓	3 (3.75%) vs. 52 (15.07%)	0.013	2 (5.71%) vs. 11 (8.73%)	0.686	35 (14.34%) vs. 17 (16.83%)	0.673	9 (9.47%) vs. 2 (6.45%)	0.926
MCH↓	0 (0.00%) vs. 24 (6.96%)	0.026	2 (5.71%) vs. 9 (7.14%)	0.932	19 (7.79%) vs. 5 (4.95%)	0.478	8 (8.42%) vs. 1 (3.23%)	0.597
RDW↑	0 (0.00%) vs. 5 (1.45%)	0.588	2 (5.71%) vs. 1 (0.79%)	0.342	5 (2.05%) vs. 0 (0.00%)	0.342	1 (1.05%) vs. 0 (0.00%)	1.000
Liver Function Tests								
ALT↑	10 (12.50%) vs. 34 (9.86%)	0.713	3 (8.57%) vs. 6 (4.76%)	0.688	24 (9.84%) vs. 10 (9.90%)	1.000	5 (5.26%) vs. 1 (3.23%)	0.942
AST↑	6 (7.50%) vs. 25 (7.25%)	1.000	3 (8.57%) vs. 6 (4.76%)	0.688	17 (6.97%) vs. 8 (7.92%)	0.934	5 (5.26%) vs. 1 (3.23%)	0.942
GGT↑	8 (10.00%) vs. 40 (11.59%)	0.724	1 (2.86%) vs. 12 (9.52%)	0.327	26 (10.66%) vs. 14 (13.86%)	0.508	7 (7.37%) vs. 5 (16.13%)	0.351
LDH↑	9 (11.25%) vs. 51 (14.78%)	0.728	5 (14.29%) vs. 19 (15.08%)	1.000	31 (12.70%) vs. 20 (19.80%)	0.128	14 (14.74%) vs. 5 (16.13%)	1.000
TBA↑	5 (6.25%) vs. 20 (5.80%)	0.769	3 (8.57%) vs. 7 (5.56%)	0.888	16 (6.56%) vs. 4 (3.96%)	0.493	4 (4.21%) vs. 3 (9.68%)	0.477
Renal Function Tests								
BUN↑	7 (8.75%) vs. 16 (4.64%)	0.439	1 (2.86%) vs. 10 (7.94%)	0.377	12 (4.92%) vs. 4 (3.96%)	0.918	7 (7.37%) vs. 3 (9.68%)	1.000
CYSC↑	15 (18.75%) vs. 92 (26.67%)	0.821	10 (28.57%) vs. 26 (20.63%)	0.335	70 (28.69%) vs. 22 (21.78%)	0.236	22 (23.16%) vs. 4 (12.90%)	0.547
UA↑	10 (12.50%) vs. 56 (16.23%)	0.561	3 (8.57%) vs. 10 (7.94%)	1.000	38 (15.57%) vs. 18 (17.82%)	0.723	7 (7.37%) vs. 3 (9.68%)	1.000

Note: Data are n (%) unless otherwise specified. Categorical variables were compared between groups using χ2 tests. *P* values less than 0.05 were considered statistically significant. The symbols ‘↓’ and ‘↑’ indicate laboratory values below and above the normal range, respectively.

Abbreviations: ALT, alanine aminotransferase; AST, aspartate aminotransferase; BUN, blood urea nitrogen; CYSC, cystatin C; EOS%, eosinophil percentage; GGT, gamma-glutamyltransferase; LDH, lactate dehydrogenase; LYM%, lymphocyte percentage; MCH, mean corpuscular hemoglobin; MONO%, monocyte percentage; NEUT%, neutrophil percentage; PLT, platelet count; RDW, red cell distribution width; TBA, total bile acid; UA, uric acid; WBC, white blood cell count.

### Clinical sequelae and laboratory abnormalities during the entire follow-up period

A total of 62.57% of SFTS survivors experienced at least one symptom, significantly higher than that of the control group (51.60%, 97/188) (**P* *< 0.05) (Table 1). Specifically, memory impairment was observed in 33.50% (401/1197) of survivors, followed by arthralgia (33.08% [396/1197], alopecia (32.25% [386/1197], and visual impairment (31.08% [372/1197] (all *P* < 0.05). These four symptoms were all significantly more prevalent among SFTS survivors compared to controls (all **P* *< 0.05). Other symptoms (including fatigue, myalgia, headache, fever, and dizziness and hearing loss) showed no significant inter-group differences (Table 1).

Regarding laboratory parameters, the most frequently observed hematologic abnormalities in SFTS survivors included decreased neutrophil percentage (NEUT%) (15.46%), followed by decreased WBC counts (12.53%), PLT counts (10.19%), lymphocyte percentage (LYM%) (10.11%), EOS% (9.44%), MCH (6.02%), monocyte percentage (MONO%) (3.84%); and elevated red cell distribution width (RDW) (1.42%). The most common liver function abnormalities were elevated LDH (19.38%), followed by gamma-glutamyltransferase (GGT) (8.60%), ALT (6.27%), AST (6.02%), TBA (5.18%); renal function test revealed elevated level of CYSC (26.48%), UA (10.44%) and BUN (6.10%). Compared with the control group, SFTS survivors exhibited significantly higher incidences of decreased EOS%, MCH and elevated LDH (all *P* < 0.05) (Table 1).

### Clinical sequelae and laboratory abnormalities in relation to patient features

In the multivariable logistic regression, SFTS survivors aged ≥60 years were significantly more likely to develop arthralgia (adjusted OR = 2.37, 95% CI: 1.22-4.97), visual impairment (adjusted OR = 2.12, 95% CI: 1.08-4.57), and increased CYSC (adjusted OR = 3.47, 95% CI: 1.45-9.65) compared to those under 40 years old. Additionally, survivors aged 40–60 years old were also at a higher risk for visual impairment and decreased NEUT% compared to those under 40 years old (all *P* < 0.05) (S2 Table).

Female sex, pre-existing medical conditions and severe-critical infection during the acute infection were identified as significant risk factors for developing long-term sequalae. For example, individuals who experienced in-hospital encephalitis had a significantly higher risk of memory impairment (44.44% [40/90] vs. 28.89% [26/90], adjusted OR = 2.39, 95% CI: 1.27-4.54) and thrombocytopenia (20.00% [18/90] vs. 6.67% [6/90], adjusted OR = 3.36, 95% CI: 1.32-9.75) compared to those without encephalitis (all *P* < 0.05) (S2 and S3 Tables). SFTS survivors with in-hospital hemorrhagic complications were at a higher risk of memory impairment (42.50% [136/320] vs. 33.75% [108/320], adjusted OR = 1.49, 95% CI: 1.07-2.07), visual impairment (40.00% [128/320] vs. 31.88% [102/320], adjusted OR = 1.45, 95% CI: 1.04-2.03); and decreased MCH (7.50% [24/320] vs. 3.13% [10/320], adjusted OR = 2.12, 95% CI: 1.05-4.49) (all *P* < 0.05) compared to those without hemorrhagic complications (S4 Table).

**Fig 2:**
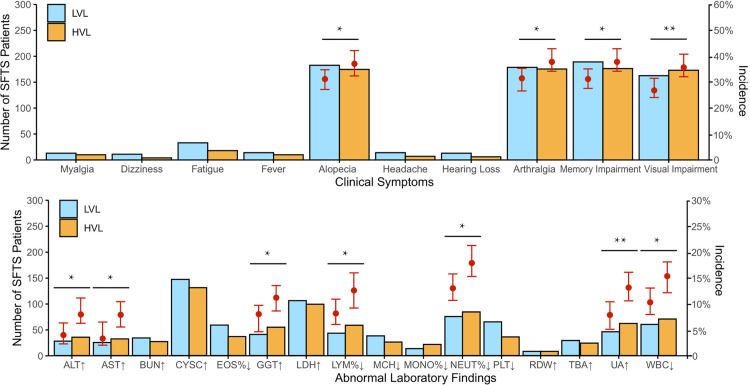
The development of clinical sequelae in the SFTSV-LVL and HVL groups during the whole follow-up period. The LVL group refered to patients with a viral load ≤ 1 × 10^6^ copies/mL on admission; the HVL group refered to patients with a viral load **≥** 1 × 10^6^ copies/mL on admission. The significant difference was obtained by using Chi-square test, with asterisks (*) denoting statistical significance. Symbols ‘↓’ and ‘↑’ denote lab values below and above normal, respectively.

SFTS survivors in the high viral load group (HVL) exhibited significantly higher incidences of alopecia, arthralgia, memory impairment, and visual impairment; as well as more frequent laboratory abnormalities, including elevated level of ALT, AST, GGT, decreased level of LYM%, NEUT%, UA, and WBC, compared to the LVL group (all *P* < 0.05) (Fig 2).

### Clinical symptoms and laboratory abnormalities in comparison with controls

**Fig 3:**
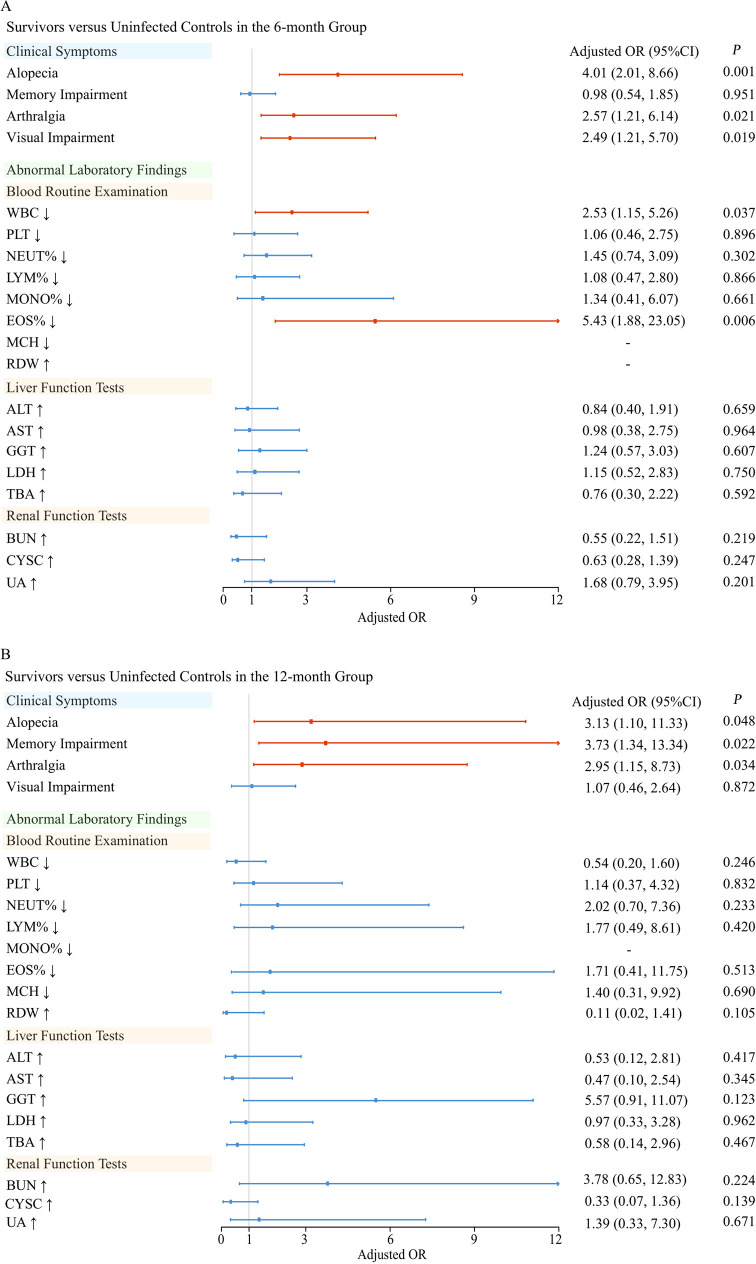
The odds ratios of sequelae between survivors and controls. **(A)** The odds ratios of sequelae between survivors and controls in the 6-month group. The 6-month group refered to patients who were followed up within 6 months or between 6-8 months after the onset. **(B)** The odds ratios of sequelae between survivors and controls in the 12-month group. The 12-month group refered to patients who were followed up between 8 and 12 months after the onset. The dot indicates the odds ratio (OR), the line segment the confidence interval, and the vertical gray line an OR of 1. Calculated by logistic regression, ORs and *P* values adjusted for age, sex, delay from disease onset, underlying diseases, with *P* < 0.05 for significance. Symbols ‘↓’ and ‘↑’ denote lab values below and above normal, respectively. Abbreviations: ALT, alanine aminotransferase; AST, aspartate aminotransferase; BUN, blood urea nitrogen; CYSC, cystatin C; EOS%, eosinophil percentage; GGT, gamma-glutamyltransferase; LDH, lactate dehydrogenase; LYM%, lymphocyte percentage; MCH, mean corpuscular hemoglobin; MONO%, monocyte percentage; NEUT%, neutrophil percentage; PLT, platelet count; RDW, red cell distribution width-coefficient of variation; TBA, total bile acid; UA, uric acid; WBC, white blood cell count.

At the 6-month follow-up, SFTS survivors demonstrated markedly elevated risks of persistent clinical sequelae compared to controls, with hair loss (33.33% vs. 13.75%; adjusted OR=4.01, 95% CI 2.01–8.66), arthralgia (24.64% vs. 10.00%; adjusted OR=2.57, 1.21–6.14), and visual decline (24.06% vs. 11.25%; adjusted OR=2.49, 1.21–5.70) showing significant associations (all **P* *< 0.05) (Fig 3A and Table 2). Concurrently, survivors exhibited higher frequencies of hematological abnormalities including leukopenia (15.65% vs. 5.00%; adjusted OR=2.53, 1.15–5.26) and eosinophil depletion (15.07% vs. 3.75%; adjusted OR=5.43, 1.88–23.05), though memory impairment prevalence showed no intergroup difference.

By 12-month post follow-up, neurological and musculoskeletal sequelae became more pronounced in survivors, with memory impairment emerging as a new significant deficit (31.75% vs. 11.43%; adjusted OR=3.73, 1.34–13.34) alongside persistent hair loss (29.37% vs. 8.57%; adjusted OR=3.13, 1.10–11.33) and worsening arthralgia (37.30% vs. 17.14%; adjusted OR=2.95, 1.15–8.73) (all **P* *< 0.05) (Fig 3B). Notably, initial hematological abnormalities resolved by this timepoint, showing no significant differences in leukocyte or eosinophil parameters relative to controls.

Longitudinal analysis revealed progressive attenuation of clinical disparities: at 18- and 24-month follow-ups, neither symptomatic sequelae nor laboratory abnormalities differed significantly between groups (Tables 2, S5 and S6 and Fig 3B). This temporal pattern suggests partial recovery from acute-phase complications, though a subset of survivors retained persistent neurological and dermatological manifestations through the first post-infection year.

### Long-term medication effects and clinical sequelae dynamics

Propensity-matched analysis of hospitalization therapies based on age, gender, and underlying conditions, revealed differential impacts on post-SFTS sequelae. Favipiravir demonstrated protective effect, with its usage significantly associated with reduced risks of alopecia (adjusted OR=0.56, 95% CI 0.30–0.89), memory impairment (adjusted OR=0.53, 95% CI 0.32–0.89), visual impairment (adjusted OR=0.49, 95% CI 0.29–0.82), and decreased EOS% (adjusted OR=0.34, 95% CI 0.15–0.73) compared to untreated controls (**P* *< 0.05; S7 Table). Conversely, corticosteroid administration was associated with increased risks of arthralgia (adjusted OR=2.17, 95% CI 1.12–5.37) and elevated BUN (adjusted OR=3.87, 95% CI 1.17–12.05), while granulocyte colony-stimulating factor (G-CSF) was associated with mitigated leukopenia (adjusted OR=0.29, 95% CI 0.11–0.89) and lactate dehydrogenase elevation (adjusted OR=0.32, 95% CI 0.14–0.68) (**P* *< 0.05; S8 and S9 Tables). Ribavirin showed no significant therapeutic or adverse associations (S10 Table).

Longitudinal tracking of 294 survivors with multiple follow-ups over 11 years revealed evolving sequelae patterns. At 6 months post-onset, hair loss, arthralgia, memory impairment and visual impairment each affected ~50% of cases. By 12 months, memory dysfunction emerged as the predominant complaint (57.9%), with other symptoms persisting at 52.6–55.3%. Symptom prevalence declined to <40% at 18 months, though residual memory (36.8%) and visual impairment (42.1%) remained detectable at 24 months (S11 Table). Heatmap analysis identified symptom clustering through Year 7, with isolated visual impairment persisting to Year 11.

Laboratory abnormalities exhibited prolonged resolution timelines: hematologic parameters (WBC, platelet counts, NEUT%, LYM%, MONO%, EOS% and MCH) and hepatic markers (ALT, AST, GGT) normalized progressively between Years 6–9. However, the abnormal PLT, LDH, TBA, and CYSC persisted even at the 10-year after disease onset. By the 11th year, all abnormal laboratory indicators had resolved (S1 Fig). This biphasic recovery pattern – rapid symptom attenuation versus gradual biomarker normalization – underscores distinct pathophysiological mechanisms governing acute versus chronic SFTS manifestations.

## Discussion

This longitudinal study provides the first comprehensive characterization of persistent clinical sequelae in SFTS survivors, revealing multi-organ impacts extending beyond acute infection. Our findings demonstrate that over 50% of patients experience prolonged convalescence marked by neurocognitive (memory impairment), musculoskeletal (arthralgia, myalgia), sensory (visual impairment) and dermatological (hair loss) manifestations – all significantly elevated compared to age-matched non-SFTS controls. These findings redefine SFTS not merely as an acute febrile illness but as a chronic viral syndrome with lasting quality-of-life implications, paralleling emerging patterns observed in post-COVID conditions [23].

The spectrum of laboratory abnormalities – spanning hematopoietic (reduced NEUT%, WBC, PLT, eosinophils), hepatic (elevated ALT, AST, LDH), and renal (increased BUN, cystatin C) parameters – mirrors acute-phase dysregulation but persists for years post-infection. Notably, the chronic level of these biomarkers directly correlated with acute disease severity [13,24], suggesting shared pathophysiology between acute viral pathogenesis and long-term damage. This pattern aligns with observations in Ebola survivors showing persistent decreased MCH or elevated ALT and AST, markers of unresolved cellular injury [25], though SFTS exhibits broader multi-organ involvement. Central to our findings is the prognostic value of acute-phase viral load, which outperformed neurological/hemorrhagic complications in predicting sequelae diversity. Patients with baseline viral loads >1 × 10^6^ copies/mL developed more long-term symptoms than those with lower titers.

As is well known, the multisystemic sequelae observed in SFTS survivors may originate from acute-phase immunopathological damage. SFTSV infection triggers a cytokine storm (e.g., elevated IL-6, IL-10, TNF-α), resulting in direct vascular endothelial damage and increased permeability, which contribute to thrombocytopenia and multiorgan dysfunction [26]. SFTSV can also invade the central nervous system, evidenced by detection of viral RNA in cerebrospinal fluid (CSF) during acute infection [26]. Thus high viral loads can effectively predict central neurological complications through both direct neuroinvasion and immunepathology [27]. Favipiravir alleviates sequelae by suppressing acute viral replication, thus mitigating pathogenic cascades. As previously demonstrated, favipiravir reduces the SFTSV load during acute infection [28], reducing direct cytopathic damage and the downstream immunopathological cascades that drive multiorgan dysfunction, accounting for the observed reduction in alopecia, memory impairment, and visual deficits among treated patients. Recent studies have shown that eosinophils also possess immunoregulatory and antiviral functions [29,30]. While its association with viral infection, including SARS-CoV and SFTSV, has been previously established [24,31], its chronic persistence suggests sustained dysfunction, a phenomenon distinct from COVID-19-associated anemia patterns [32]. Conversely, research on the relationship between MCH and viral infections remains limited, with exception of its negative correlation with the severity of COVID-19 as recently demonstrated [33]. Elevated LDH, a marker of cellular injury, demonstrated a significant biological gradient with viremia in Ebola survivors (27% increase per log10 viral load, **P* *= 0.002), indicating direct virus-associated tissue damage [34].

The chronic sequelae observed in SFTS survivors share overlapping spectrum yet different patterns when contextualized within the broader spectrum of viral hemorrhagic fevers. Neurocognitive deficits (memory impairment) and persistent arthralgia in SFTS mirror sequelae documented in Ebola survivors, where musculoskeletal pain and ocular complications frequently persist for years post-infection [35]. However, SFTS exhibits a unique multisystem chronicity profile: while Crimean-Congo hemorrhagic fever survivors report transient autonomic dysfunction (alopecia, anorexia) and sensory deficits [36], SFTS patients demonstrate prolonged visual impairment and hematological dysregulation – manifestations more aligned with chronic post-Lassa hearing loss [37] and dengue-related ocular complications [38]. Notably, the predominance of memory impairment in SFTS (31.75% prevalence at 12 months) contrasts with the 15.9% rates observed in Dengue virus survivors [39], suggesting potential neurotropic mechanisms distinct to SFTSV pathogenesis.

This divergence may reflect both viral-specific pathophysiology and demographic influences. The older age profile of SFTS patients (median 65 years vs. < 60 years in most hemorrhagic fever cohorts [37,40,41]) may amplify symptom chronicity, potentially through mechanisms such as age-related immunosenescence and comorbidities. For instance, while chikungunya-associated arthralgia typically resolves within 2 years [42], its persistence in SFTS survivors may stem from synergistic viral-endothelial interactions exacerbated by vascular aging [43,44]. Similarly, the decade-long thrombocytopenia observed in our cohort exceeds Lassa fever recovery timelines [45], possibly reflecting SFTSV’s unique megakaryocyte tropism combined with age-impaired hematopoiesis [46]. These findings underscore the critical interplay between viral pathogenesis and host biology in shaping long-term outcomes.

We also observed abnormal laboratory indicators that exhibited trends opposite to those observed during the acute phase. For example, at 6-months of follow-up, SFTS survivors demonstrated higher prevalence of decreased RDW and decreased LDH compared to controls (S12-S14 Tables). These inverse patterns, though statistically significant, exhibited limited clinical relevance given their low frequency (≤5% of the cohort) and transient nature, prompting their exclusion from primary analysis.

A significant strength of our study lies in its large patient sample size, which was evaluated over an extended period through the follow-up of two cohorts in two hospitals. Nevertheless, we recognize that the generalizability of our findings may be restricted by the study’s regional focus (Henan, China) and the fact that the cohorts were predominantly composed of elderly females. Although key pathophysiological insights, such as viral load as a predictor of sequelae, are likely to be generalized, we advise exercising caution when extrapolating the exact symptom prevalence or treatment effects to different settings. Future multi-country cohort studies will contribute to clarifying the global applicability of these findings. Despite these limitations, our findings provide critical insights into the long-term impacts on the quality of life and mental health status of SFTS survivors. Future research conducted across multiple independent medical institutions is essential to corroborate the present findings and to elucidate the mechanisms responsible for the development of long-term SFTS sequelae.

## Supporting information

S1 Table Summary of participants’ demographics and clinical features. (DOCX)

S2 Table Association of age, sex, and comorbidities with the risk of sequelae. (DOCX)

S3 Table Comparison of sequelae between encephalitis and non-encephalitis patients among SFTS survivors. (DOCX)

S4 Table Comparison of sequelae between hemorrhagic and non-hemorrhagic patients among SFTS survivors. (DOCX)

S5 Table Prevalence of clinical symptoms and laboratory findings on physical examination. (DOCX)

S6 Table The odds ratios of sequelae in SFTS survivors and controls at 18 and 24 months, and between severe and mild cases. (DOCX)

S7 Table Comparison of sequelae in SFTS survivors based on favipiravir treatment during the acute phase. (DOCX)

S8 Table Comparison of sequelae in SFTS survivors based on corticosteroids treatment during the acute phase. (DOCX)

S9 Table Comparison of sequelae in SFTS survivors based on recombinant human granulocyte colony-stimulating factor treatment during the acute phase. (DOCX)

S10 Table Comparison of sequelae in SFTS survivors based on ribavirin treatment during the acute phase. (DOCX)

S11 Table The dynamic change of clinical sequelae in patients with multiple follow-ups within two years. (DOCX)

S12 Table Abnormal laboratory indicators in participants that showed trends opposite to those observed during the acute phase throughout the entire follow-up period. (DOCX)

S13 Table Abnormal laboratory indicators in participants showed trends opposite to those observed during the acute phase at the 6-month and 12-month follow-up time points. (DOCX)

S14 Table Abnormal laboratory indicators in participants showed trends opposite to those observed during the acute phase at the 18-month and 24-month follow-up time points. (DOCX)

S1 Fig The dynamic change of clinical sequelae in patients with multiple follow-ups at different time points. The heatmap uses color intensity to represent the number of individuals within each category, with darker colors indicating a higher number of individuals. The symbols ‘↓’ and ‘↑’ indicate laboratory values below and above the normal range, respectively. Abbreviations: BUN, blood urea nitrogen; CYSC, cystatin C; EOS%, eosinophil percentage; GGT, gamma-glutamyltransferase; LDH, lactate dehydrogenase; LYM%, lymphocyte percentage; MCH, mean corpuscular hemoglobin; MONO%, monocyte percentage; NEUT%, neutrophil percentage; PLT, platelet count; RDW, red cell distribution width; UA, uric acid; WBC, white blood cell count.
(TIF)

S1 File SFTS sequelae assessment questionnaire. (DOCX)
