# Supplementary material for: Long-term clinical sequelae in severe fever with thrombocytopenia syndrome: A longitudinal cohort study
Source: PLoS Negl Trop Dis. 2025 Aug 12;19(8):e0013276. doi: 10.1371/journal.pntd.0013276 (PMC12360653; doi:10.1371/journal.pntd.0013276)
Supplement: S1 Table — (DOCX) [file pntd.0013276.s001.docx]

| **S1 Table. Summary of participants' demographics and clinical features.** | | | | |
| --- | --- | --- | --- | --- |
| **Characteristics** | **Total Participants** | **Non-recruited** | **Recruited** | ***P* value** |
|  | **(N=3875)** | **(N=2678)** | **(N=1197)** |  |
| **Demographic Characteristics** |  |  |  |  |
| Gender, Female: n (%) | 2396 (61.83%) | 1639 (61.20%) | 757 (63.24%) | 0.241 |
| Age at Onset, years | 63 (54, 71) | 64 (55, 71) | 62 (52, 69) | 0.358 |
| **Time Interval (Median, Interquartile Range)** | | | | |
| Days from Onset to Hospital Admission | 5 (4, 6) | 5 (4, 7) | 5 (3, 6) | 0.131 |
| Days from Hospital Admission to Discharge | 8 (6, 10) | 8 (6, 10) | 9 (7, 11) | 0.325 |
| **Comorbidities, n (%)** | | | | |
| Any Comorbidity | 1663 (42.92%) | 1173 (43.80%) | 490 (40.94%) | 0.103 |
| Hypertension | 602 (15.54%) | 435 (16.24%) | 167 (13.95%) | 0.076 |
| Diabetes | 269 (6.94%) | 185 (6.91%) | 84 (7.02%) | 0.956 |
| Chronic Obstructive Pulmonary Disease | 205 (5.29%) | 139 (5.19%) | 66 (5.51%) | 0.736 |
| Hepatitis | 271 (6.99%) | 174 (6.50%) | 97 (8.10%) | 0.081 |
| Cardiovascular Disease | 186 (4.80%) | 120 (4.48%) | 66 (5.51%) | 0.191 |
| Severe cases | 1382 (35.66%) | 929 (34.69%) | 453 (37.84%) | 0.071 |
| Note: Data are n (%) unless otherwise specified. Categorical variables were compared between groups using χ2 tests. Continuous variables were presented as medians with interquartile ranges (IQR) and were compared using the Mann-Whitney U test. *P* values less than 0.05 were considered statistically significant. | | | | |
