# Supplementary material for: Long-term clinical sequelae in severe fever with thrombocytopenia syndrome: A longitudinal cohort study
Source: PLoS Negl Trop Dis. 2025 Aug 12;19(8):e0013276. doi: 10.1371/journal.pntd.0013276 (PMC12360653; doi:10.1371/journal.pntd.0013276)
Supplement: S2 Table — (DOCX) [file pntd.0013276.s002.docx]

| **S2 Table. Association of age, sex, and comorbidities with the risk of sequelae.** | | |
| --- | --- | --- |
| **Characteristics** | **OR (95% CI)** | ***P* value** |
| **Sequelae** |  |  |
| Male | Reference |  |
| Female | 1.70 (1.27, 2.26) | <0.001^†^ |
| **Sequelae** |  |  |
| Non-comorbidity | Reference |  |
| Any-comorbidity | 2.55 (1.87, 3.51) | <0.001^*^ |
| **Alopecia** |  |  |
| ≤40 years old | Reference |  |
| 40-60 years old | 1.16 (0.64, 2.14) | 0.628^#^ |
| ≥60 years old | 0.62 (0.34, 1.16) | 0.125^#^ |
| **Memory Impairment** |  |  |
| ≤40 years old | Reference |  |
| 40-60 years old | 1.80 (0.97, 3.52) | 0.073^#^ |
| ≥60 years old | 1.53 (0.82, 2.99) | 0.194^#^ |
| **Arthralgia** |  |  |
| ≤40 years old | Reference |  |
| 40-60 years old | 1.96 (1.01, 4.09) | 0.058^#^ |
| ≥60 years old | 2.37 (1.22, 4.97) | 0.015^#^ |
| **Visual Impairment** |  |  |
| ≤40 years old | Reference |  |
| 40-60 years old | 2.40 (1.22, 5.17) | 0.017^#^ |
| ≥60 years old | 2.12 (1.08, 4.57) | 0.040^#^ |
| **WBC↓** |  |  |
| ≤40 years old | Reference |  |
| 40-60 years old | 2.09 (0.82, 7.10) | 0.169^#^ |
| ≥60 years old | 2.06 (0.80, 7.03) | 0.181^#^ |
| **PLT↓** |  |  |
| ≤40 years old | Reference |  |
| 40-60 years old | 2.46 (0.72, 15.35) | 0.224^#^ |
| ≥60 years old | 3.21 (0.96, 19.99) | 0.113^#^ |
| **NEUT%↓** |  |  |
| ≤40 years old | Reference |  |
| 40-60 years old | 3.96 (1.41, 16.54) | 0.024^#^ |
| ≥60 years old | 3.14 (0.96, 13.16) | 0.061^#^ |
| **LYM%↓** |  |  |
| ≤40 years old | Reference |  |
| 40-60 years old | 0.76 (0.33, 2.09) | 0.560^#^ |
| ≥60 years old | 0.89 (0.38, 2.41) | 0.795^#^ |
| **MONO%↓** |  |  |
| ≤40 years old | Reference |  |
| 40-60 years old | 2.60 (0.53, 47.20) | 0.355^#^ |
| ≥60 years old | 1.15 (0.22, 21.30) | 0.892^#^ |
| **EOS%↓** |  |  |
| ≤40 years old | Reference |  |
| 40-60 years old | 0.82 (0.35, 2.25) | 0.673^#^ |
| ≥60 years old | 0.63 (0.27, 1.75) | 0.330^#^ |
| **MCH↓** |  |  |
| ≤40 years old | Reference |  |
| 40-60 years old | 0.94 (0.38, 2.83) | 0.896^#^ |
| ≥60 years old | 0.29 (0.11, 0.94) | 0.023^#^ |
| **RDW↑** |  |  |
| ≤40 years old | Reference |  |
| 40-60 years old | 1.48 (0.28, 27.56) | 0.710^#^ |
| ≥60 years old | 0.32 (0.04, 6.69) | 0.336^#^ |
| **ALT↑** |  |  |
| ≤40 years old | Reference |  |
| 40-60 years old | 1.03 (0.39, 3.55) | 0.957^#^ |
| ≥60 years old | 0.51 (0.19, 1.80) | 0.234^#^ |
| **GGT↑** |  |  |
| ≤40 years old | Reference |  |
| 40-60 years old | 1.30 (0.44, 5.54) | 0.678^#^ |
| ≥60 years old | 1.59 (0.55, 6.72) | 0.451^#^ |
| **LDH↑** |  |  |
| ≤40 years old | Reference |  |
| 40-60 years old | 1.12 (0.49, 3.06) | 0.799^#^ |
| ≥60 years old | 2.15 (0.95, 5.79) | 0.090^#^ |
| **TBA↑** |  |  |
| ≤40 years old | Reference |  |
| 40-60 years old | 1.35 (0.38, 8.63) | 0.690^#^ |
| ≥60 years old | 1.22 (0.34, 7.79) | 0.790^#^ |
| **UA↑** |  |  |
| ≤40 years old | Reference |  |
| 40-60 years old | 0.60 (0.22, 1.92) | 0.343^#^ |
| ≥60 years old | 1.24 (0.49, 3.81) | 0.675^#^ |
| **CYSC↑** |  |  |
| ≤40 years old | Reference |  |
| 40-60 years old | 1.83 (0.76, 5.12) | 0.205^#^ |
| ≥60 years old | 3.47 (1.45, 9.65) | 0.009^#^ |
| Note: ORs and *P* values were calculated by logistic regression model. ‘†’indicates that the adjustment factors were age, underlying diseases, and admission delay. ‘*’indicates that the adjustment factors were age, sex, and admission delay. ‘#’indicates that the adjustment factors were sex, underlying diseases, and admission delay. *P* values less than 0.05 were considered statistically significant. The symbols ‘↓’ and ‘↑’ indicate laboratory values below and above the normal range, respectively. Abbreviations: ALT, alanine aminotransferase; CYSC, cystatin C; EOS%, eosinophil percentage; GGT, gamma-glutamyltransferase; LDH, lactate dehydrogenase; LYM%, lymphocyte percentage; MCH, mean corpuscular hemoglobin; MONO%, monocyte percentage; NEUT%, neutrophil percentage; PLT, platelet count; RDW, red cell distribution width; TBA, total bile acid; UA, uric acid; WBC, white blood cell count. | | |
