# Supplementary material for: Long-term clinical sequelae in severe fever with thrombocytopenia syndrome: A longitudinal cohort study
Source: PLoS Negl Trop Dis. 2025 Aug 12;19(8):e0013276. doi: 10.1371/journal.pntd.0013276 (PMC12360653; doi:10.1371/journal.pntd.0013276)
Supplement: S3 Table — (DOCX) [file pntd.0013276.s003.docx]

| **S3 Table. Comparison of sequelae between encephalitis and non-encephalitis patients among SFTS survivors.** | | | | |
| --- | --- | --- | --- | --- |
| **Sequelae** | **Non-encephalitis patients  (N=90)** | **Encephalitis patients  (N=90)** | **OR (95% CI)** | ***P* value** |
| **Clinical Symptoms** |  |  |  |  |
| Alopecia | 33（36.67%） | 36（40.00%） | 1.36 (0.73, 2.55) | 0.331 |
| Memory Impairment | 26（28.89%） | 40（44.44%） | 2.39 (1.27, 4.54) | 0.007 |
| Arthralgia | 38（42.22%） | 36（40.00%） | 1.08 (0.58, 2.01) | 0.803 |
| Visual Impairment | 30（33.33%） | 30（33.33%） | 1.16 (0.61, 2.20) | 0.644 |
| **Abnormal Laboratory Findings** |  |  |  |  |
| **Blood Routine Examination** |  |  |  |  |
| WBC↓ | 8（8.89%） | 14（15.56%） | 1.81 (0.73, 4.80) | 0.209 |
| PLT↓ | 6（6.67%） | 18（20.00%） | 3.36 (1.32, 9.75) | 0.016 |
| NEUT%↓ | 15（16.67%） | 16（17.78%） | 1.03 (0.47, 2.27) | 0.941 |
| LYM%↓ | 5（5.56%） | 10（11.11%） | 2.02 (0.68, 6.75) | 0.219 |
| MONO%↓ | 3（3.33%） | 3（3.33%） | 0.95 (0.17, 5.25) | 0.948 |
| EOS%↓ | 5（5.56%） | 9（10.00%） | 1.80 (0.59, 6.08) | 0.314 |
| MCH↓ | 6（6.67%） | 4（4.44%） | 0.62 (0.15, 2.26) | 0.476 |
| RDW↑ | 3（3.33%） | 0（0.00%） | - | - |
| **Liver Function Tests** |  |  |  |  |
| ALT↑ | 10（11.11%） | 6（6.67%） | 0.57 (0.18, 1.62) | 0.302 |
| AST↑ | 11（12.22%） | 3（3.33%） | 0.25 (0.05, 1.05) | 0.053 |
| GGT↑ | 13（14.44%） | 7（7.78%） | 0.49 (0.17, 1.29) | 0.161 |
| LDH↑ | 16（17.78%） | 15（16.67%） | 0.87 (0.39, 1.93) | 0.734 |
| TBA↑ | 7（7.78%） | 5（5.56%） | 0.66 (0.19, 2.16) | 0.491 |
| **Renal Function Tests** |  |  |  |  |
| BUN↑ | 10（11.11%） | 3（3.33%） | 0.30 (0.06, 1.03) | 0.077 |
| CYSC↑ | 17（18.89%） | 19（21.11%） | 0.78 (0.34, 1.80) | 0.559 |
| UA↑ | 8（8.89%） | 8（8.89%） | 0.81 (0.27, 2.43) | 0.700 |

Note: Data are n (%) unless otherwise specified. Propensity score matching (PSM) with a 1:1 ratio was used to match baseline characteristics such as age, sex between the two groups. ORs and *P* values were calculated by logistic regression model. Confounders such as age, sex, delay from disease onset, underlying diseases were adjusted. *P* values less than 0.05 were considered statistically significant. The symbols '↓' and '↑' indicate laboratory values below and above the normal range, respectively.

Abbreviations: ALT, alanine aminotransferase; AST, aspartate aminotransferase; BUN, blood urea nitrogen; CYSC, cystatin C; EOS%, eosinophil percentage; GGT, gamma-glutamyltransferase; LDH, lactate dehydrogenase; LYM%, lymphocyte percentage; MCH, mean corpuscular hemoglobin; MONO%, monocyte percentage; NEUT%, neutrophil percentage; PLT, platelet count; RDW, red cell distribution width; TBA, total bile acid; UA, uric acid; WBC, white blood cell count.
