# Supplementary material for: Long-term clinical sequelae in severe fever with thrombocytopenia syndrome: A longitudinal cohort study
Source: PLoS Negl Trop Dis. 2025 Aug 12;19(8):e0013276. doi: 10.1371/journal.pntd.0013276 (PMC12360653; doi:10.1371/journal.pntd.0013276)
Supplement: S4 Table — (DOCX) [file pntd.0013276.s004.docx]

| **S4 Table. Comparison of sequelae between hemorrhagic and non-hemorrhagic patients among SFTS survivors.** | | | | |
| --- | --- | --- | --- | --- |
| **Sequelae** | **Non-hemorrhagic patients  (N=320)** | **Hemorrhagic patients  (N=320)** | **OR (95% CI)** | ***P* value** |
| **Clinical Symptoms** |  |  |  |  |
| Alopecia | 107（33.44%） | 113（35.31%） | 1.09 (0.77, 1.55) | 0.622 |
| Memory Impairment | 108（33.75%） | 136（42.50%） | 1.49 (1.07, 2.07) | 0.017 |
| Arthralgia | 114（35.63%） | 128（40.00%） | 1.22 (0.88, 1.69) | 0.241 |
| Visual Impairment | 102（31.88%） | 128（40.00%） | 1.45 (1.04, 2.03) | 0.027 |
| **Abnormal Laboratory Findings** |  |  |  |  |
| **Blood Routine Examination** |  |  |  |  |
| WBC↓ | 43（13.44%） | 37（11.56%） | 0.84 (0.52, 1.34) | 0.459 |
| PLT↓ | 33（10.31%） | 31（9.69%） | 0.92 (0.55, 1.55) | 0.766 |
| NEUT%↓ | 48（15.00%） | 56（17.50%） | 1.19 (0.78, 1.83) | 0.410 |
| LYM%↓ | 30（9.38%） | 38（11.88%） | 1.29 (0.78, 2.17) | 0.321 |
| MONO%↓ | 14（4.38%） | 14（4.38%） | 1.00 (0.46, 2.16) | 0.995 |
| EOS%↓ | 24（7.50%） | 31（9.69%） | 1.32 (0.76, 2.33) | 0.331 |
| MCH↓ | 10（3.13%） | 24（7.50%） | 2.12 (1.05, 4.49) | 0.041 |
| RDW↑ | 5（1.56%） | 7（2.19%） | 1.49 (0.46, 5.17) | 0.509 |
| **Liver Function Tests** |  |  |  |  |
| ALT↑ | 18（5.63%） | 18（5.63%） | 1.02 (0.52, 2.01) | 0.954 |
| AST↑ | 25（7.81%） | 19（5.94%） | 0.76 (0.40, 1.41) | 0.385 |
| GGT↑ | 30（9.38%） | 27（8.44%） | 0.91 (0.52, 1.57) | 0.732 |
| LDH↑ | 62（19.38%） | 70（21.88%） | 1.29 (0.87, 1.91) | 0.210 |
| TBA↑ | 18（5.63%） | 13（4.06%） | 0.75 (0.35, 1.55) | 0.442 |
| **Renal Function Tests** |  |  |  |  |
| BUN↑ | 16（5.00%） | 24（7.50%） | 1.50 (0.77, 2.97) | 0.235 |
| CYSC↑ | 66（20.63%） | 72（22.50%） | 1.07 (0.69, 1.66) | 0.769 |
| UA↑ | 27（8.44%） | 31（9.69%） | 1.03 (0.58, 1.83) | 0.924 |

Note: Data are n (%) unless otherwise specified. Propensity score matching (PSM) with a 1:1 ratio was used to match baseline characteristics such as age, sex between the two groups. ORs and *P* values were calculated by logistic regression model. Confounders such as age, sex, delay from disease onset, underlying diseases were adjusted. *P* values less than 0.05 were considered statistically significant. The symbols '↓' and '↑' indicate laboratory values below and above the normal range, respectively.

Abbreviations: ALT, alanine aminotransferase; AST, aspartate aminotransferase; BUN, blood urea nitrogen; CYSC, cystatin C; EOS%, eosinophil percentage; GGT, gamma-glutamyltransferase; LDH, lactate dehydrogenase; LYM%, lymphocyte percentage; MCH, mean corpuscular hemoglobin; MONO%, monocyte percentage; NEUT%, neutrophil percentage; PLT, platelet count; RDW, red cell distribution width; TBA, total bile acid; UA, uric acid; WBC, white blood cell count.
