# Supplementary material for: Long-term clinical sequelae in severe fever with thrombocytopenia syndrome: A longitudinal cohort study
Source: PLoS Negl Trop Dis. 2025 Aug 12;19(8):e0013276. doi: 10.1371/journal.pntd.0013276 (PMC12360653; doi:10.1371/journal.pntd.0013276)
Supplement: S5 Table — (DOCX) [file pntd.0013276.s005.docx]

| **S5 Table. Prevalence of clinical symptoms and laboratory findings on physical examination.** | | | | | | | | |
| --- | --- | --- | --- | --- | --- | --- | --- | --- |
| **Characteristics** | **Uninfected Controls vs. SFTS Survivors** | | | | **Mild Cases vs. Severe Cases** | | | |
|  | **18-month** |  | **24-month** |  | **18-month** |  | **24-month** |  |
| Total Number per Group | 4 vs. 80 |  | 12 vs. 61 |  | 55 vs. 25 |  | 43 vs. 18 |  |
| **Clinical Symptoms** |  | *P* value |  | *P* value |  | *P* value |  | *P* value |
| Alopecia | 0（0.00%） vs. 19（23.75%） | 0.646 | 2（16.67%） vs. 12（19.67%） | 0.879 | 16（29.09%） vs. 3（12.00%） | 0.166 | 9（20.93%） vs. 3（16.67%） | 0.694 |
| Memory Impairment | 0（0.00%） vs. 23（28.75%） | 0.506 | 0（0.00%） vs. 11（18.03%） | 0.248 | 17（30.91%） vs. 6（24.00%） | 0.568 | 9（20.93%） vs. 2（11.11%） | 0.116 |
| Arthralgia | 0（0.00%） vs. 22（27.50%） | 0.531 | 1（8.33%） vs. 9（14.75%） | 0.895 | 18（32.72%） vs. 4（16.00%） | 0.128 | 6（13.95%） vs. 3（16.67%） | 1.000 |
| Visual Impairment | 0（0.00%） vs. 21（26.25%） | 0.564 | 3（25.00%） vs. 13（21.31%） | 0.932 | 17（30.91%） vs. 4（16.00%） | 0.111 | 11（25.58%） vs. 2（11.11%） | 0.320 |
| **Abnormal Laboratory Findings** | | | | | | | | |
| **Blood Routine Examination** |  |  |  |  |  |  |  |  |
| WBC↓ | 1（25.00%） vs. 14（17.50%） | 1.000 | 3（25.00%） vs. 3（4.92%） | 0.082 | 12（21.82%） vs. 2（8.00%） | 0.248 | 1（2.33%） vs. 1（5.56%） | 1.000 |
| PLT↓ | 0（0.00%） vs. 7（8.75%） | 1.000 | 2（16.67%） vs. 5（8.20%） | 0.736 | 5（9.09%） vs. 2（8.00%） | 1.000 | 3（6.98%） vs. 2（11.11%） | 0.880 |
| NEUT%↓ | 0（0.00%） vs. 15（18.75%） | 0.759 | 4（33.33%） vs. 11（18.03%） | 0.454 | 10（18.18%） vs. 5（20.00%） | 1.000 | 8（18.60%） vs. 3（16.67%） | 1.000 |
| LYM%↓ | 0（0.00%） vs. 9（11.25%） | 1.000 | 0（0.00%） vs. 4（6.56%） | 0.809 | 6（10.91%） vs. 3（12.00%） | 1.000 | 4（9.30%） vs. 0（0.00%） | 0.496 |
| MONO%↓ | 0（0.00%） vs. 2（2.50%） | 1.000 | 0（0.00%） vs. 1（1.64%） | 1.000 | 1（1.82%） vs. 1（4.00%） | 1.000 | 1（2.33%） vs. 0（0.00%） | 1.000 |
| EOS%↓ | 1（25.00%） vs. 4（5.00%） | 0.583 | 0（0.00%） vs. 6（9.84%） | 0.558 | 1（1.82%） vs. 3（12.00%） | 0.158 | 5（11.63%） vs. 1（5.56%） | 0.902 |
| MCH↓ | 0（0.00%） vs. 4（5.00%） | 1.000 | 1（8.33%） vs. 2（3.28%） | 1.000 | 3（5.45%） vs. 1（4.00%） | 1.000 | 2（4.65%） vs. 0（0.00%） | 0.945 |
| RDW↑ | 0（0.00%） vs. 1（1.25%） | 1.000 | 0（0.00%） vs. 1（1.64%） | 1.000 | 1（1.82%） vs. 0（0.00%） | 1.000 | 1（2.33%） vs. 0（0.00%） | 1.000 |
| **Liver Function Tests** |  |  |  |  |  |  |  |  |
| ALT↑ | 0（0.00%） vs. 1（1.25%） | 1.000 | 0（0.00%） vs. 5（8.20%） | 0.725 | 1（1.82%） vs. 0（0.00%） | 1.000 | 4（9.30%） vs. 1（5.56%） | 1.000 |
| AST↑ | 0（0.00%） vs. 2（2.50%） | 1.000 | 0（0.00%） vs. 4（6.56%） | 0.865 | 2（3.64%） vs. 0（0.00%） | 0.829 | 3（6.98%） vs. 1（5.56%） | 1.000 |
| GGT↑ | 0（0.00%） vs. 5（6.25%） | 1.000 | 2（16.67%） vs. 8（13.11%） | 1.000 | 2（3.64%） vs. 3（12.00%） | 0.374 | 6（13.95%） vs. 2（11.11%） | 1.000 |
| LDH↑ | 0（0.00%） vs. 23（28.75%） | 0.851 | 4（33.33%） vs. 19（31.15%） | 1.000 | 18（32.73%） vs. 5（20.00%） | 0.402 | 12（27.91%） vs. 7（38.89%） | 0.865 |
| TBA↑ | 0（0.00%） vs. 5（6.25%） | 1.000 | 1（8.33%） vs. 2（3.28%） | 1.000 | 4（7.27%） vs. 1（4.00%） | 0.978 | 0（0.00%） vs. 2（11.11%） | 0.177 |
| **Renal Function Tests** |  |  |  |  |  |  |  |  |
| BUN↑ | 0（0.00%） vs. 6（7.50%） | 1.000 | 1（8.33%） vs. 5（8.20%） | 1.000 | 3（5.45%） vs. 3（12.00%） | 0.771 | 3（6.98%） vs. 2（11.11%） | 1.000 |
| CYSC↑ | 0（0.00%） vs. 30（37.50%） | 0.837 | 1（8.33%） vs. 16（26.23%） | 0.831 | 18（32.73%） vs. 12（48.00%） | 1.000 | 14（32.56%） vs. 2（11.11%） | 0.881 |
| UA↑ | 1（25.00%） vs. 8（10.00%） | 0.955 | 3（25.00%） vs. 8（13.11%） | 0.769 | 4（7.27%） vs. 4（16.00%） | 0.638 | 6（13.95%） vs. 2（11.11%） | 1.000 |
| Note: Data are n (%) unless otherwise specified. Categorical variables were compared between groups using χ2 tests. *P* values less than 0.05 were considered statistically significant. The symbols '↓' and '↑' indicate laboratory values below and above the normal range, respectively. Abbreviations: ALT, alanine aminotransferase; AST, aspartate aminotransferase; BUN, blood urea nitrogen; CYSC, cystatin C; EOS%, eosinophil percentage; GGT, gamma-glutamyltransferase; LDH, lactate dehydrogenase; LYM%, lymphocyte percentage; MCH, mean corpuscular hemoglobin; MONO%, monocyte percentage; NEUT%, neutrophil percentage; PLT, platelet count; RDW, red cell distribution width; TBA, total bile acid; UA, uric acid; WBC, white blood cell count. | | | | | | | | |
