# Supplementary material for: Long-term clinical sequelae in severe fever with thrombocytopenia syndrome: A longitudinal cohort study
Source: PLoS Negl Trop Dis. 2025 Aug 12;19(8):e0013276. doi: 10.1371/journal.pntd.0013276 (PMC12360653; doi:10.1371/journal.pntd.0013276)
Supplement: S6 Table — (DOCX) [file pntd.0013276.s006.docx]

| **S6 Table. The odds ratios of sequelae in SFTS survivors and controls at 18 and 24 months, and between severe and mild cases.** | | | | | | | | |
| --- | --- | --- | --- | --- | --- | --- | --- | --- |
| **Sequelae** | **SFTS Survivors vs. Controls  at the 18-month point** | | **SFTS Survivors vs. Controls  at the 24-month point** | | **Severe Cases vs. Mild Cases at the 18-month point** | | **Severe Cases vs. Mild Cases at the 24-month point** | |
|  | **OR (95% CI)** | ***P* value** | **OR (95% CI)** | ***P* value** | **OR (95% CI)** | ***P* value** | **OR (95% CI)** | ***P* value** |
| **Clinical Symptoms** |  |  |  |  |  |  |  |  |
| Alopecia | - | 0.646* | 3.52 (0.72, 26.86) | 0.155 | 0.49 (0.12, 1.68) | 0.273 | 0.90 (0.21, 3.64) | 0.881 |
| Memory Impairment | - | 0.506* | - | 0.248* | 0.69 (0.21, 2.09) | 0.515 | 0.13 (0.01, 1.09) | 0.052 |
| Arthralgia | - | 0.531* | 3.74 (0.54, 77.12) | 0.254 | 0.49 (0.14, 1.56) | 0.237 | 2.59 (0.50, 13.92) | 0.250 |
| Visual Impairment | - | 0.564* | 0.76 (0.36, 7.63) | 0.552 | 0.34 (0.09, 1.18) | 0.102 | 0.47 (0.10, 1.90) | 0.309 |
| **Abnormal Laboratory Findings** |  |  |  |  |  |  |  |  |
| **Blood Routine Examination** |  |  |  |  |  |  |  |  |
| WBC↓ | 0.89 (0.06, 24.70) | 0.933 | 0.04 (0.00, 1.19) | 0.054 | 0.25 (0.03, 1.20) | 0.118 | - | 1.000* |
| PLT↓ | - | 1.000* | 0.28 (0.03, 2.89) | 0.259 | 0.80 (0.09, 5.23) | 0.824 | - | 0.880* |
| NEUT%↓ | - | 0.759* | 0.73 (0.16, 3.58) | 0.683 | 2.11 (0.51, 9.03) | 0.297 | 0.82 (0.13, 4.33) | 0.823 |
| LYM%↓ | - | 1.000* | - | 0.809* | 0.92 (0.16, 4.45) | 0.920 | - | 0.496* |
| MONO%↓ | - | 1.000* | - | 1.000* | - | 1.000* | - | 1.000* |
| EOS%↓ | 0.10 (0.01, 4.35) | 0.208 | - | 0.558* | - | 0.158* | 1.02 (0.04, 14.36) | 0.987 |
| MCH↓ | - | 1.000* | 2.38 (0.10, 196.60) | 0.623 | 1.21 (0.05, 13.74) | 0.885 | - | 0.945* |
| RDW↑ | - | 1.000* | - | 1.000* | - | 1.000* | - | 1.000* |
| **Liver Function Tests** |  |  |  |  |  |  |  |  |
| ALT↑ | - | 1.000* | - | 0.725* |  | 1.000 | 1.75 (0.07, 30.64) | 0.694 |
| AST↑ | - | 1.000* | - | 0.865* |  | 0.998 | 1.06 (0.04, 14.45) | 0.966 |
| GGT↑ | - | 1.000* | 0.41 (0.05, 3.75) | 0.394 | 4.52 (0.57, 47.70) | 0.161 | 0.54 (0.06, 3.36) | 0.533 |
| LDH↑ | - | 0.851* | 0.78 (0.15, 4.15) | 0.764 | 0.19 (0.04, 1.17) | 0.052 | 0.75 (0.17, 2.90) | 0.684 |
| TBA↑ | - | 1.000* | 0.58 (0.01, 65.05) | 0.774 | 0.23 (0.01, 2.38) | 0.290 |  |  |
| **Renal Function Tests** |  |  |  |  |  |  |  |  |
| BUN↑ | - | 1.000* | 0.90 (0.06, 22.16) | 0.936 | 0.95 (0.09, 10.10) | 0.966 | 1.07 (0.06, 18.17) | 0.960 |
| CYSC↑ | - | 0.837* | 0.98 (0.07, 26.52) | 0.991 | 0.85 (0.19, 3.70) | 0.825 | 0.26 (0.01, 3.36) | 0.336 |
| UA↑ | 0.14 (0.03, 5.67) | 0.262 | 0.32 (0.04, 2.39) | 0.259 | 1.26 (0.22, 6.94) | 0.787 | 4.08 (0.31, 60.33) | 0.273 |
| Note: ORs and *P* values were calculated by logistic regression model. Confounders such as age, sex, delay from disease onset, underlying diseases were adjusted. *P* values less than 0.05 were considered statistically significant. The symbols '↓' and '↑' indicate laboratory values below and above the normal range, respectively. '*' represents the *P* values obtained from χ2 tests comparing categorical variables between groups. Abbreviations: ALT, alanine aminotransferase; AST, aspartate aminotransferase; BUN, blood urea nitrogen; CYSC, cystatin C; EOS%, eosinophil percentage; GGT, gamma-glutamyltransferase; LDH, lactate dehydrogenase; LYM%, lymphocyte percentage; MCH, mean corpuscular hemoglobin; MONO%, monocyte percentage; NEUT%, neutrophil percentage; PLT, platelet count; RDW, red cell distribution width; TBA, total bile acid; UA, uric acid; WBC, white blood cell count. | | | | | | | | |
