# Supplementary material for: Long-term clinical sequelae in severe fever with thrombocytopenia syndrome: A longitudinal cohort study
Source: PLoS Negl Trop Dis. 2025 Aug 12;19(8):e0013276. doi: 10.1371/journal.pntd.0013276 (PMC12360653; doi:10.1371/journal.pntd.0013276)
Supplement: S7 Table — (DOCX) [file pntd.0013276.s007.docx]

| **S7 Table. Comparison of sequelae in SFTS survivors based on favipiravir treatment**  **during the acute phase.** | | | | |
| --- | --- | --- | --- | --- |
| **Sequelae** | **Non-FPV (N=151)** | **FPV (N=151)** | **OR (95% CI)** | ***P* value** |
| **Clinical Symptoms** |  |  |  |  |
| Alopecia | 51（33.77%） | 35（23.18%） | 0.56 (0.30, 0.89) | 0.017 |
| Memory Impairment | 57（37.75%） | 40（26.49%） | 0.53 (0.32, 0.89) | 0.016 |
| Arthralgia | 51（33.77%） | 53（35.10%） | 0.99 (0.60, 1.64) | 0.958 |
| Visual Impairment | 57（37.75%） | 39（25.83%） | 0.49 (0.29, 0.82) | 0.006 |
| **Abnormal Laboratory Findings** |  |  |  |  |
| **Blood Routine Examination** |  |  |  |  |
| WBC↓ | 30（19.87%） | 22（14.57%） | 0.60 (0.32, 1.12) | 0.111 |
| PLT↓ | 22（14.57%） | 19（12.58%） | 0.78 (0.39, 1.54) | 0.472 |
| NEUT%↓ | 27（17.88%） | 19（12.58%） | 0.60 (0.31, 1.14) | 0.122 |
| LYM%↓ | 20（13.25%） | 16（10.60%） | 0.70 (0.34, 1.43) | 0.326 |
| MONO%↓ | 10（6.62%） | 6（3.97%） | 0.56 (0.18, 1.62) | 0.299 |
| EOS%↓ | 25（16.56%） | 10（6.62%） | 0.34 (0.15, 0.73) | 0.007 |
| MCH↓ | 16（10.60%） | 11（7.28%） | 0.64 (0.27, 1.44) | 0.285 |
| RDW↑ | 2（1.32%） | 3（1.99%） | 1.46 (0.23, 1.16) | 0.690 |
| **Liver Function Tests** |  |  |  |  |
| ALT↑ | 11（7.28%） | 14（9.27%） | 1.24 (0.54, 2.93) | 0.618 |
| AST↑ | 10（6.62%） | 14（9.27%） | 1.40 (0.60, 3.42) | 0.440 |
| GGT↑ | 18（11.92%） | 14（9.27%） | 0.74 (0.34, 1.57) | 0.438 |
| LDH↑ | 23（15.23%） | 28（18.54%） | 1.18 (0.62, 2.27) | 0.608 |
| TBA↑ | 8（5.30%） | 8（5.30%） | 0.91 (0.32, 2.62) | 0.855 |
| **Renal Function Tests** |  |  |  |  |
| BUN↑ | 11（7.28%） | 9（5.96%） | 0.86 (0.32, 2.24) | 0.752 |
| CYSC↑ | 46（30.46%） | 40（26.49%） | 0.86 (0.47, 1.55) | 0.612 |
| UA↑ | 15（9.93%） | 18（11.92%） | 1.34 (0.60, 3.05) | 0.472 |

Note: Data are n (%) unless otherwise specified. FPV denoted SFTS patients who were treated with favipiravir during the acute phase, while Non-FPV denoted those who were not. Propensity score matching (PSM) with a 1:1 ratio was used to match baseline characteristics such as age, sex, and underlying diseases between the two groups. ORs and *P* values were calculated by logistic regression model. Confounders such as age, sex, delay from disease onset, underlying diseases were adjusted. *P* values less than 0.05 were considered statistically significant. The symbols '↓' and '↑' indicate laboratory values below and above the normal range, respectively.
Abbreviations: ALT, alanine aminotransferase; AST, aspartate aminotransferase; BUN, blood urea nitrogen; CYSC, cystatin C; EOS%, eosinophil percentage; GGT, gamma-glutamyltransferase; LDH, lactate dehydrogenase; LYM%, lymphocyte percentage; MCH, mean corpuscular hemoglobin; MONO%, monocyte percentage; NEUT%, neutrophil percentage; PLT, platelet count; RDW, red cell distribution width; TBA, total bile acid; UA, uric acid; WBC, white blood cell count.
