# Supplementary material for: Long-term clinical sequelae in severe fever with thrombocytopenia syndrome: A longitudinal cohort study
Source: PLoS Negl Trop Dis. 2025 Aug 12;19(8):e0013276. doi: 10.1371/journal.pntd.0013276 (PMC12360653; doi:10.1371/journal.pntd.0013276)
Supplement: S8 Table — (DOCX) [file pntd.0013276.s008.docx]

| **S8 Table. Comparison of sequelae in SFTS survivors based on corticosteroids treatment during the acute phase.** | | | | |
| --- | --- | --- | --- | --- |
| **Sequelae** | **Non-CS (N=70)** | **CS**  **(N=70)** | **OR (95% CI)** | ***P* value** |
| **Clinical Symptoms** |  |  |  |  |
| Alopecia | 23（32.86%） | 19（27.14%） | 0.69 (0.32, 1.50) | 0.353 |
| Memory Impairment | 24（34.29%） | 23（32.86%） | 0.89 (0.43, 1.84) | 0.754 |
| Arthralgia | 15（21.43%） | 27（38.57%） | 2.17 (1.12, 5.37) | 0.037 |
| Visual Impairment | 24（34.29%） | 20（28.57%） | 0.71 (0.33, 1.51) | 0.369 |
| **Abnormal Laboratory Findings** |  |  |  |  |
| **Blood Routine Examination** |  |  |  |  |
| WBC↓ | 7（10.00%） | 12（17.14%） | 1.87 (0.69, 5.37) | 0.225 |
| PLT↓ | 9（12.86%） | 5（7.14%） | 0.52 (0.15, 1.58) | 0.259 |
| NEUT%↓ | 14（20.00%） | 19（27.14%） | 1.50 (0.68, 3.40) | 0.320 |
| LYM%↓ | 7（10.00%） | 8（11.43%） | 1.23 (0.41, 3.83) | 0.709 |
| MONO%↓ | 3（4.29%） | 3（4.29%） | 0.96 (0.16, 5.62) | 0.960 |
| EOS%↓ | 3（4.29%） | 9（12.86%） | 3.26 (0.92, 15.27) | 0.087 |
| MCH↓ | 4（5.71%） | 2（2.86%） | 0.41 (0.05, 2.33) | 0.335 |
| RDW↑ | 0（4.29%） | 0（4.29%） | - | - |
| **Liver Function Tests** |  |  |  |  |
| ALT↑ | 5（7.14%） | 5（7.14%） | 0.93 (0.24, 3.63) | 0.918 |
| AST↑ | 2（2.86%） | 5（7.14%） | 2.55 (0.52, 18.40) | 0.275 |
| GGT↑ | 10（14.29%） | 5（7.14%） | 0.39 (0.10, 1.28) | 0.132 |
| LDH↑ | 6（8.57%） | 11（15.71%） | 1.82 (0.63, 5.76) | 0.282 |
| TBA↑ | 2（2.86%） | 8（11.43%） | 4.10 (0.95, 28.44) | 0.087 |
| **Renal Function Tests** |  |  |  |  |
| BUN↑ | 3（4.29%） | 12（17.14%） | 3.87 (1.17, 12.05) | 0.017 |
| CYSC↑ | 18（25.71%） | 12（17.14%） | 0.45 (0.16, 1.17) | 0.105 |
| UA↑ | 12（17.14%） | 10（14.29%） | 0.76 (0.27, 2.16) | 0.612 |

Note: Data are n (%) unless otherwise specified. CS denoted SFTS patients who were treated with corticosteroids during the acute phase, while Non-CS denoted those who were not. Propensity score matching (PSM) with a 1:1 ratio was used to match baseline characteristics such as age, sex, and underlying diseases between the two groups. ORs and *P* values were calculated by logistic regression model. Confounders such as age, sex, delay from disease onset, underlying diseases were adjusted. *P* values less than 0.05 were considered statistically significant. The symbols '↓' and '↑' indicate laboratory values below and above the normal range, respectively.
Abbreviations: ALT, alanine aminotransferase; AST, aspartate aminotransferase; BUN, blood urea nitrogen; CYSC, cystatin C; EOS%, eosinophil percentage; GGT, gamma-glutamyltransferase; LDH, lactate dehydrogenase; LYM%, lymphocyte percentage; MCH, mean corpuscular hemoglobin; MONO%, monocyte percentage; NEUT%, neutrophil percentage; PLT, platelet count; RDW, red cell distribution width; TBA, total bile acid; UA, uric acid; WBC, white blood cell count.
