# Supplementary material for: Long-term clinical sequelae in severe fever with thrombocytopenia syndrome: A longitudinal cohort study
Source: PLoS Negl Trop Dis. 2025 Aug 12;19(8):e0013276. doi: 10.1371/journal.pntd.0013276 (PMC12360653; doi:10.1371/journal.pntd.0013276)
Supplement: S9 Table — (DOCX) [file pntd.0013276.s009.docx]

| **S9 Table. Comparison of sequelae in SFTS survivors based on recombinant human granulocyte colony-stimulating factor treatment during the acute phase.** | | | | |
| --- | --- | --- | --- | --- |
| **Sequelae** | **Non-RhG-CSF (N=107)** | **RhG-CSF (N=107)** | **OR (95% CI)** | ***P*** value |
| **Clinical Symptoms** |  |  |  |  |
| Alopecia | 28（26.17%） | 39（36.45%） | 1.33 (0.72, 2.48) | 0.360 |
| Memory Impairment | 26（24.30%） | 42（39.25%） | 1.71 (0.93, 3.16) | 0.085 |
| Arthralgia | 37（34.58%） | 46（42.99%） | 1.12 (0.63, 1.99) | 0.702 |
| Visual Impairment | 31（28.97%） | 46（42.99%） | 1.47 (0.82, 2.65) | 0.195 |
| **Abnormal Laboratory Findings** |  |  |  |  |
| **Blood Routine Examination** |  |  |  |  |
| WBC↓ | 16（14.95%） | 6（5.61%） | 0.29 (0.11, 0.89) | 0.037 |
| PLT↓ | 7（6.54%） | 8（7.48%） | 1.12 (0.38, 3.36) | 0.837 |
| NEUT%↓ | 14（13.08%） | 14（13.08%） | 0.94 (0.42, 2.13) | 0.890 |
| LYM%↓ | 5（4.67%） | 9（8.41%） | 1.87 (0.61, 6.38) | 0.288 |
| MONO%↓ | 1（0.93%） | 2（1.87%） | 2.05 (0.18, 46.05) | 0.571 |
| EOS%↓ | 8（7.48%） | 7（6.54%） | 0.82 (0.27, 2.49) | 0.727 |
| MCH↓ | 3（2.80%） | 3（2.80%） | 0.96 (0.17, 5.45) | 0.958 |
| RDW↑ | 1（0.93%） | 0（0.00%） | - | 0.984 |
| **Liver Function Tests** |  |  |  |  |
| ALT↑ | 4（3.74%） | 8（7.48%） | 2.00 (0.60, 7.77) | 0.275 |
| AST↑ | 5（4.67%） | 5（4.67%） | 0.95 (0.26, 3.56) | 0.943 |
| GGT↑ | 10（9.35%） | 6（5.61%） | 0.53 (0.17, 1.53) | 0.251 |
| LDH↑ | 29（27.10%） | 12（11.21%） | 0.32 (0.14, 0.68) | 0.005 |
| TBA↑ | 10（9.35%） | 8（7.48%） | 0.71 (0.26, 1.90) | 0.495 |
| **Renal Function Tests** |  |  |  |  |
| BUN↑ | 4（3.74%） | 5（4.67%） | 1.30 (0.33, 5.49) | 0.705 |
| CYSC↑ | 19（17.76%） | 10（9.35%） | 0.44 (0.17, 1.07) | 0.075 |
| UA↑ | 9（8.41%） | 8（7.48%） | 0.84 (0.27, 2.58) | 0.759 |

Note: Data are n (%) unless otherwise specified. RhG-CSF denoted SFTS patients who were treated with recombinant human granulocyte colony-stimulating factor during the acute phase, while Non-RhG-CSF denoted those who were not. Propensity score matching (PSM) with a 1:1 ratio was used to match baseline characteristics such as age, sex, and underlying diseases between the two groups. ORs and *P* values were calculated by logistic regression model. Confounders such as age, sex, delay from disease onset, underlying diseases were adjusted. *P* values less than 0.05 were considered statistically significant. The symbols '↓' and '↑' indicate laboratory values below and above the normal range, respectively.
Abbreviations: ALT, alanine aminotransferase; AST, aspartate aminotransferase; BUN, blood urea nitrogen; CYSC, cystatin C; EOS%, eosinophil percentage; GGT, gamma-glutamyltransferase; LDH, lactate dehydrogenase; LYM%, lymphocyte percentage; MCH, mean corpuscular hemoglobin; MONO%, monocyte percentage; NEUT%, neutrophil percentage; PLT, platelet count; RDW, red cell distribution width; TBA, total bile acid; UA, uric acid; WBC, white blood cell count.
