# Supplementary material for: Long-term clinical sequelae in severe fever with thrombocytopenia syndrome: A longitudinal cohort study
Source: PLoS Negl Trop Dis. 2025 Aug 12;19(8):e0013276. doi: 10.1371/journal.pntd.0013276 (PMC12360653; doi:10.1371/journal.pntd.0013276)
Supplement: S10 Table — (DOCX) [file pntd.0013276.s010.docx]

| **S10 Table. Comparison of sequelae in SFTS survivors based on ribavirin treatment during the acute phase.** | | | | |
| --- | --- | --- | --- | --- |
| **Sequelae** | **Non-RBV (N=20)** | **RBV (N=20)** | **OR (95% CI)** | ***P* value** |
| **Clinical Symptoms** |  |  |  |  |
| Alopecia | 5（25.00%） | 7（35.00%） | 0.99 (0.23, 4.36) | 0.997 |
| Memory Impairment | 6（30.00%） | 6（30.00%） | 0.53 (0.10, 2.50) | 0.424 |
| Arthralgia | 6（30.00%） | 6（30.00%） | 0.53 (0.10, 2.50) | 0.424 |
| Visual Impairment | 5（25.00%） | 5（25.00%） | 0.60 (0.12, 2.87) | 0.515 |
| **Abnormal Laboratory Findings** |  |  |  |  |
| **Blood Routine Examination** |  |  |  |  |
| WBC↓ | 7（35.00%） | 4（20.00%） | 0.53 (0.19, 1.49) | 0.412 |
| PLT↓ | 3（15.00%） | 0（0.00%） | - | 0.230* |
| NEUT%↓ | 5（25.00%） | 1（5.00%） | 0.11 (0.02, 0.97) | 0.084 |
| LYM%↓ | 2（10.00%） | 0（0.00%） | - | 0.468* |
| MONO%↓ | 1（5.00%） | 0（0.00%） | - | 1.000* |
| EOS%↓ | 2（10.00%） | 1（5.00%） | 0.35 (0.01, 4.23) | 0.422 |
| MCH↓ | 1（5.00%） | 1（5.00%） | 1.00 (0.03, 9.51) | 1.000 |
| RDW↑ | 0（0.00%） | 0（0.00%） | - | - |
| **Liver Function Tests** |  |  |  |  |
| ALT↑ | 1（5.00%） | 0（0.00%） | - | 1.000* |
| AST↑ | 0（0.00%） | 1（5.00%） | - | 1.000* |
| GGT↑ | 2（10.00%） | 2（10.00%） | 0.87 (0.06, 11.77) | 0.908 |
| LDH↑ | 2（10.00%） | 1（5.00%） | 0.35 (0.01, 4.23) | 0.422 |
| TBA↑ | 2（10.00%） | 0（0.00%） | - | 0.468* |
| **Renal Function Tests** |  |  |  |  |
| BUN↑ | 1（5.00%） | 2（10.00%） | 3.08 (0.16, 15.27) | 0.472 |
| CYSC↑ | 2（10.00%） | 4（20.00%） | 2.79 (0.23, 9.27) | 0.997 |
| UA↑ | 0（0.00%） | 3（15.00%） | - | 0.998* |

Note: Data are n (%) unless otherwise specified. RBV denoted SFTS patients who were treated with ribavirin during the acute phase, while Non-RBV denoted those who were not. Propensity score matching (PSM) with a 1:1 ratio was used to match baseline characteristics such as age, sex, and underlying diseases between the two groups. ORs and *P* values were calculated by logistic regression model. Confounders such as age, sex, delay from disease onset, underlying diseases were adjusted. *P* values less than 0.05 were considered statistically significant. The symbols '↓' and '↑' indicate laboratory values below and above the normal range, respectively. '*' represents the *P* values obtained from χ2 tests comparing categorical variables between groups.
Abbreviations: ALT, alanine aminotransferase; AST, aspartate aminotransferase; BUN, blood urea nitrogen; CYSC, cystatin C; EOS%, eosinophil percentage; GGT, gamma-glutamyltransferase; LDH, lactate dehydrogenase; LYM%, lymphocyte percentage; MCH, mean corpuscular hemoglobin; MONO%, monocyte percentage; NEUT%, neutrophil percentage; PLT, platelet count; RDW, red cell distribution width; TBA, total bile acid; UA, uric acid; WBC, white blood cell count.
