# Supplementary material for: Long-term clinical sequelae in severe fever with thrombocytopenia syndrome: A longitudinal cohort study
Source: PLoS Negl Trop Dis. 2025 Aug 12;19(8):e0013276. doi: 10.1371/journal.pntd.0013276 (PMC12360653; doi:10.1371/journal.pntd.0013276)
Supplement: S11 Table — (DOCX) [file pntd.0013276.s011.docx]

| **S11 Table. The dynamic change of clinical sequelae in patients with multiple follow-ups within two years.** | | | | |
| --- | --- | --- | --- | --- |
| **Sequelae** | **Time Point** | | | |
|  | **6-month** | **12-month** | **18-month** | **24-month** |
| Total Number per Group | 113 | 38 | 36 | 19 |
| **Clinical Symptoms** |  |  |  |  |
| Alopecia | 61（53.98%） | 22（57.89%） | 12（33.33%） | 7（36.84%） |
| Memory Impairment | 54（47.79%） | 21（55.26%） | 14（38.88%） | 6（31.58%） |
| Arthralgia | 48（42.48%） | 20（52.63%） | 13（36.11%） | 3（15.79%） |
| Visual Impairment | 49（43.36%） | 20（52.63%） | 12（33.33%） | 8（42.11%） |
| **Abnormal Laboratory Findings** | | | | |
| **Blood Routine Examination** |  |  |  |  |
| WBC↓ | 30（26.55%） | 5（13.16%） | 8（22.22%） | 1（5.26%） |
| PLT↓ | 21（18.58%） | 8（21.05%） | 8（22.22%） | 4（21.05%） |
| NEUT%↓ | 28（24.78%） | 9（23.68%） | 8（22.22%） | 3（15.79%） |
| LYM%↓ | 21（18.58%） | 8（21.05%） | 7（19.44%） | 3（15.79%） |
| MONO%↓ | 12（10.62%） | 4（10.53%） | 6（16.67%） | 1（5.26%） |
| EOS%↓ | 24（21.24%） | 7（18.42%） | 6（16.67%） | 3（15.79%） |
| MCH↓ | 16（14.16%） | 5（13.16%） | 7（19.44%） | 2（10.53%） |
| RDW↑ | 1（0.88%） | 1（2.63%） | 0（0.00%） | 0（0.00%） |
| **Liver Function Tests** |  |  |  |  |
| ALT↑ | 7（6.19%） | 0（0.00%） | 1（2.78%） | 3（15.79%） |
| AST↑ | 9（7.96%） | 2（5.26%） | 0（0.00%） | 0（0.00%） |
| GGT↑ | 11（9.73%） | 1（2.63%） | 4（11.11%） | 1（5.26%） |
| LDH↑ | 20（17.70%） | 5（13.16%） | 8（22.22%） | 5（26.32%） |
| TBA↑ | 5（4.42%） | 2（5.26%） | 3（8.33%） | 1（5.26%） |
| **Renal Function Tests** |  |  |  |  |
| BUN↑ | 9（7.96%） | 3（7.89%） | 3（8.33%） | 4（21.05%） |
| CYSC↑ | 18（15.93%） | 7（18.42%） | 9（25.00%） | 8（42.11%） |
| UA↑ | 8（7.08%） | 2（5.26%） | 4（11.11%） | 1（5.26%） |
| Note: Data are n (%) unless otherwise specified. The symbols '↓' and '↑' indicate laboratory values below and above the normal range, respectively. Abbreviations: ALT, alanine aminotransferase; AST, aspartate aminotransferase; BUN, blood urea nitrogen; CYSC, cystatin C; EOS%, eosinophil percentage; GGT, gamma-glutamyltransferase; LDH, lactate dehydrogenase; LYM%, lymphocyte percentage; MCH, mean corpuscular hemoglobin; MONO%, monocyte percentage; NEUT%, neutrophil percentage; PLT, platelet count; RDW, red cell distribution width; TBA, total bile acid; UA, uric acid; WBC, white blood cell count. | | | | |
