# Supplementary material for: Long-term clinical sequelae in severe fever with thrombocytopenia syndrome: A longitudinal cohort study
Source: PLoS Negl Trop Dis. 2025 Aug 12;19(8):e0013276. doi: 10.1371/journal.pntd.0013276 (PMC12360653; doi:10.1371/journal.pntd.0013276)
Supplement: S12 Table — (DOCX) [file pntd.0013276.s012.docx]

| **S12 Table. Abnormal laboratory indicators in participants that showed trends opposite to those observed during the acute phase throughout the entire follow-up period.** | | | | |
| --- | --- | --- | --- | --- |
| **Sequelae** | **Total Population** | **Uninfected Controls** | **SFTS Survivors** | ***P* value** |
|  | **(N=1385)** | **(N=188)** | **(N=1197)** |  |
| WBC↑ | 13（0.94%） | 3（1.60%） | 10（0.84%） | 0.574 |
| PLT↑ | 42（3.03%） | 1（0.53%） | 41（3.43%） | 0.061 |
| NEUT%↑ | 182（13.14%） | 21（11.17%） | 161（13.45%） | 0.388 |
| LYM%↑ | 222（16.03%） | 33（17.55%） | 189（15.79%） | 0.716 |
| MONO%↑ | 125（9.03%） | 12（6.38%） | 113（9.44%） | 0.189 |
| EOS%↑ | 99 (7.15%) | 10 (5.32%) | 89 (7.44%) | 0.329 |
| MCH↑ | 281（20.29%） | 34（18.09%） | 247（20.63%） | 0.385 |
| RDW↓ | 46（3.32%） | 1（0.53%） | 45（3.76%） | 0.032 |
| GGT↓ | 19（1.37%） | 1（0.53%） | 18（1.50%） | 0.473 |
| LDH↓ | 42（3.03%） | 1（0.53%） | 41（3.43%） | 0.061 |
| BUN↓ | 168（12.13%） | 17（9.04%） | 151（12.61%） | 0.213 |
| CYSC↓ | 150（10.83%） | 17（9.04%） | 133（11.11%） | 0.472 |
| UA↓ | 254（18.34%） | 25（13.30%） | 229（19.13%） | 0.071 |
| Note: Data are n (%) unless otherwise specified. Categorical variables were compared between groups using χ2 tests. *P* values less than 0.05 were considered statistically significant. The symbols '↓' and '↑' indicate laboratory values below and above the normal range, respectively. Abbreviations: BUN, blood urea nitrogen; CYSC, cystatin C; EOS%, eosinophil percentage; GGT, gamma-glutamyltransferase; LDH, lactate dehydrogenase; LYM%, lymphocyte percentage; MCH, mean corpuscular hemoglobin; MONO%, monocyte percentage; NEUT%, neutrophil percentage; PLT, platelet count; RDW, red cell distribution width; UA, uric acid; WBC, white blood cell count. | | | | |
