# Supplementary material for: Long-term clinical sequelae in severe fever with thrombocytopenia syndrome: A longitudinal cohort study
Source: PLoS Negl Trop Dis. 2025 Aug 12;19(8):e0013276. doi: 10.1371/journal.pntd.0013276 (PMC12360653; doi:10.1371/journal.pntd.0013276)
Supplement: S13 Table — (DOCX) [file pntd.0013276.s013.docx]

| **S13 Table. Abnormal laboratory indicators in participants showed trends opposite to those observed during the acute phase at the 6-month and 12-month follow-up time points.** | | | | | | | | |
| --- | --- | --- | --- | --- | --- | --- | --- | --- |
| **Sequelae** | **Uninfected Controls vs. SFTS Survivors** | | | | **Mild Cases vs. Severe Cases** | | | |
|  | **6-month** |  | **12-month** |  | **6-month** |  | **12-month** |  |
| Total Number per Group | 80 vs. 345 | *P* value | 35 vs. 126 | *P* value | 244 vs. 101 | *P* value | 95 vs. 31 | *P* value |
| WBC↑ | 2 (2.50%) vs. 5 (1.45%) | 0.897 | 0 (0.00%) vs. 2 (1.59%) | 1.000 | 3 (1.23%) vs. 2 (1.98%) | 0.964 | 0 (0.00%) vs. 2 (6.45%) | 0.087 |
| PLT↑ | 0 (0.00%) vs. 20 (5.80%) | 0.057 | 0 (0.00%) vs. 7 (5.56%) | 0.289 | 16 (6.56%) vs. 4 (3.96%) | 0.503 | 5 (5.26%) vs. 2 (6.45%) | 1.000 |
| NEUT%↑ | 10 (12.50%) vs. 34 (9.86%) | 0.713 | 3 (8.57%) vs. 16 (12.70%) | 0.556 | 23 (9.43%) vs. 11 (10.89%) | 0.808 | 12 (12.63%) vs. 4 (12.90%) | 1.000 |
| LYM%↑ | 16 (20.00%) vs. 64 (18.55%) | 1.000 | 7 (20.00%) vs. 20 (15.87%) | 0.968 | 46 (18.85%) vs. 18 (17.82%) | 0.971 | 15 (15.79%) vs. 5 (16.13%) | 1.000 |
| MONO%↑ | 4 (5.00%) vs. 47 (13.62%) | 0.053 | 2 (5.71%) vs. 6 (4.76%) | 1.000 | 39 (15.98%) vs. 14 (13.86%) | 0.762 | 3 (3.16%) vs. 3 (9.68%) | 0.292 |
| EOS%↑ | 3 (3.75%) vs. 23 (6.67%) | 0.421 | 3 (8.57%) vs. 4 (3.17%) | 0.447 | 19 (7.79%) vs. 4 (3.96%) | 0.297 | 3 (3.16%) vs. 1 (3.23%) | 1.000 |
| MCH↑ | 10 (12.50%) vs. 61 (17.68%) | 0.269 | 7 (20.00%) vs. 23 (18.25%) | 1.000 | 48 (19.67%) vs. 13 (12.87%) | 0.185 | 19 (20.00%) vs. 4 (12.90%) | 0.587 |
| RDW↓ | 0 (0.00%) vs. 21 (6.09%) | 0.048 | 0 (0.00%) vs. 11 (8.73%) | 0.088 | 16 (6.56%) vs. 5 (4.95%) | 0.749 | 10 (10.53%) vs. 1 (3.23%) | 0.436 |
| GGT↓ | 0 (0.00%) vs. 11 (3.19%) | 0.202 | 0 (0.00%) vs. 1 (0.79%) | 1.000 | 8 (3.28%) vs. 3 (2.97%) | 1.000 | 1 (1.05%) vs. 0 (0.00%) | 1.000 |
| LDH↓ | 0 (0.00%) vs. 27 (7.83%) | 0.021 | 1 (2.86%) vs. 4 (3.17%) | 1.000 | 18 (7.38%) vs. 9 (8.91%) | 0.825 | 4 (4.21%) vs. 0 (0.00%) | 0.521 |
| BUN↓ | 0 (0.00%) vs. 20 (5.80%) | 0.057 | 0 (0.00%) vs. 4 (3.17%) | 0.568 | 17 (6.97%) vs. 12 (11.88%) | 0.067 | 2 (2.11%) vs. 2 (6.45%) | 0.583 |
| CYSC↓ | 7 (8.75%) vs. 71 (20.58%) | 0.287 | 6 (17.14%) vs. 21 (16.67%) | 1.000 | 46 (18.85%) vs. 25 (24.75%) | 0.055 | 16 (16.84%) vs. 5 (16.13%) | 0.949 |
| UA↓ | 16 (20.00%) vs. 68 (19.71%) | 0.849 | 6 (17.14%) vs. 23 (18.25%) | 0.559 | 46 (18.85%) vs. 22 (21.78%) | 0.589 | 19 (20.00%) vs. 4 (12.90%) | 0.457 |
| Note: Data are n (%) unless otherwise specified. Categorical variables were compared between groups using χ2 tests. *P* values less than 0.05 were considered statistically significant. The symbols '↓' and '↑' indicate laboratory values below and above the normal range, respectively. Abbreviations: BUN, blood urea nitrogen; CYSC, cystatin C; EOS%, eosinophil percentage; GGT, gamma-glutamyltransferase; LDH, lactate dehydrogenase; LYM%, lymphocyte percentage; MCH, mean corpuscular hemoglobin; MONO%, monocyte percentage; NEUT%, neutrophil percentage; PLT, platelet count; RDW, red cell distribution width; UA, uric acid; WBC, white blood cell count. | | | | | | | | |
