# Supplementary material for: Long-term clinical sequelae in severe fever with thrombocytopenia syndrome: A longitudinal cohort study
Source: PLoS Negl Trop Dis. 2025 Aug 12;19(8):e0013276. doi: 10.1371/journal.pntd.0013276 (PMC12360653; doi:10.1371/journal.pntd.0013276)
Supplement: S14 Table — (DOCX) [file pntd.0013276.s014.docx]

| **S14 Table. Abnormal laboratory indicators in participants showed trends opposite to those observed during the acute phase at the 18-month and 24-month follow-up time points.** | | | | | | | | |
| --- | --- | --- | --- | --- | --- | --- | --- | --- |
| **Sequelae** | **Uninfected Controls vs. SFTS Survivors** | | | | **Mild Cases vs. Severe Cases** | | | |
|  | **18-month** |  | **24-month** |  | **18-month** |  | **24-month** |  |
| Total Number per Group | 4 vs. 80 | *P* value | 12 vs. 61 | *P* value | 55 vs. 25 | *P* value | 43 vs. 18 | *P* value |
| WBC↑ | 0 (0.00%) vs. 0 (0.00%) | - | 0 (0.00%) vs. 0 (0.00%) | - | 0 (0.00%) vs. 0 (0.00%) | - | 0 (0.00%) vs. 0 (0.00%) | - |
| PLT↑ | 0 (0.00%) vs. 3 (3.75%) | 1.000 | 0 (0.00%) vs. 1 (1.64%) | 1.000 | 3 (5.45%) vs. 0 (0.00%) | 0.589 | 1 (2.33%) vs. 0 (0.00%) | 1.000 |
| NEUT%↑ | 0 (0.00%) vs. 10 (12.50%) | 1.000 | 0 (0.00%) vs. 9 (14.75%) | 0.331 | 7 (12.73%) vs. 3 (12.00%) | 1.000 | 7 (16.28%) vs. 2 (11.11%) | 1.000 |
| LYM%↑ | 0 (0.00%) vs. 11 (13.75%) | 0.956 | 3 (25.00%) vs. 10 (16.39%) | 0.804 | 7 (12.73%) vs. 4 (16.00%) | 0.935 | 7 (16.28%) vs. 3 (16.67%) | 1.000 |
| MONO%↑ | 0 (0.00%) vs. 5 (6.25%) | 1.000 | 0 (0.00%) vs. 4 (6.56%) | 0.809 | 4 (7.27%) vs. 1 (4.00%) | 0.969 | 1 (2.33%) vs. 3 (16.67%) | 0.099 |
| EOS%↑ | 0（0.00%） vs. 10（12.50%） | 1.000 | 1（8.33%） vs. 6（9.84%） | 1.000 | 7（12.73%） vs. 3（12.00%） | 1.000 | 4（9.30%） vs. 2（11.11%） | 1.000 |
| MCH↑ | 0 (0.00%) vs. 23 (28.75%) | 0.478 | 4 (33.33%) vs. 16 (26.23%) | 0.933 | 16 (29.09%) vs. 7 (28.00%) | 1.000 | 12 (27.91%) vs. 4 (22.22%) | 1.000 |
| RDW↓ | 0 (0.00%) vs. 5 (6.25%) | 1.000 | 1 (8.33%) vs. 3 (4.92%) | 1.000 | 4 (7.27%) vs. 1 (4.00%) | 0.969 | 2 (4.65%) vs. 1 (5.56%) | 1.000 |
| GGT↓ | 0 (0.00%) vs. 0 (0.00%) | - | 0 (0.00%) vs. 0 (0.00%) | - | 0 (0.00%) vs. 0 (0.00%) | - | 0 (0.00%) vs. 0 (0.00%) | - |
| LDH↓ | 0 (0.00%) vs. 0 (0.00%) | - | 0 (0.00%) vs. 0 (0.00%) | - | 0 (0.00%) vs. 0 (0.00%) | - | 0 (0.00%) vs. 0 (0.00%) | - |
| BUN↓ | 0 (0.00%) vs. 7 (8.75%) | 1.000 | 0 (0.00%) vs. 3 (4.92%) | 0.831 | 5 (9.09%) vs. 2 (8.00%) | 0.969 | 3 (6.98%) vs. 0 (0.00%) | 0.489 |
| CYSC↓ | 1 (25.00%) vs. 9 (11.25%) | 0.449 | 3 (25.00%) vs. 8 (13.11%) | 0.098 | 6 (17.14%) vs. 3 (12.00%) | 1.000 | 5 (11.63%) vs. 3 (16.67%) | 0.209 |
| UA↓ | 0 (0.00%) vs. 18 (22.50%) | 0.581 | 0 (0.00%) vs. 9 (14.75%) | 0.242 | 11 (20.00%) vs. 7 (28.00%) | 1.000 | 7 (16.28%) vs. 2 (11.11%) | 1.000 |
| Note: Data are n (%) unless otherwise specified. Categorical variables were compared between groups using χ2 tests. *P* values less than 0.05 were considered statistically significant. The symbols '↓' and '↑' indicate laboratory values below and above the normal range, respectively. Abbreviations: BUN, blood urea nitrogen; CYSC, cystatin C; EOS%, eosinophil percentage; GGT, gamma-glutamyltransferase; LDH, lactate dehydrogenase; LYM%, lymphocyte percentage; MCH, mean corpuscular hemoglobin; MONO%, monocyte percentage; NEUT%, neutrophil percentage; PLT, platelet count; RDW, red cell distribution width; UA, uric acid; WBC, white blood cell count. | | | | | | | | |
