# Supplementary figures and images for: Long-term clinical sequelae in severe fever with thrombocytopenia syndrome: A longitudinal cohort study
Source: PLoS Negl Trop Dis. 2025 Aug 12;19(8):e0013276. doi: 10.1371/journal.pntd.0013276 (PMC12360653; doi:10.1371/journal.pntd.0013276)

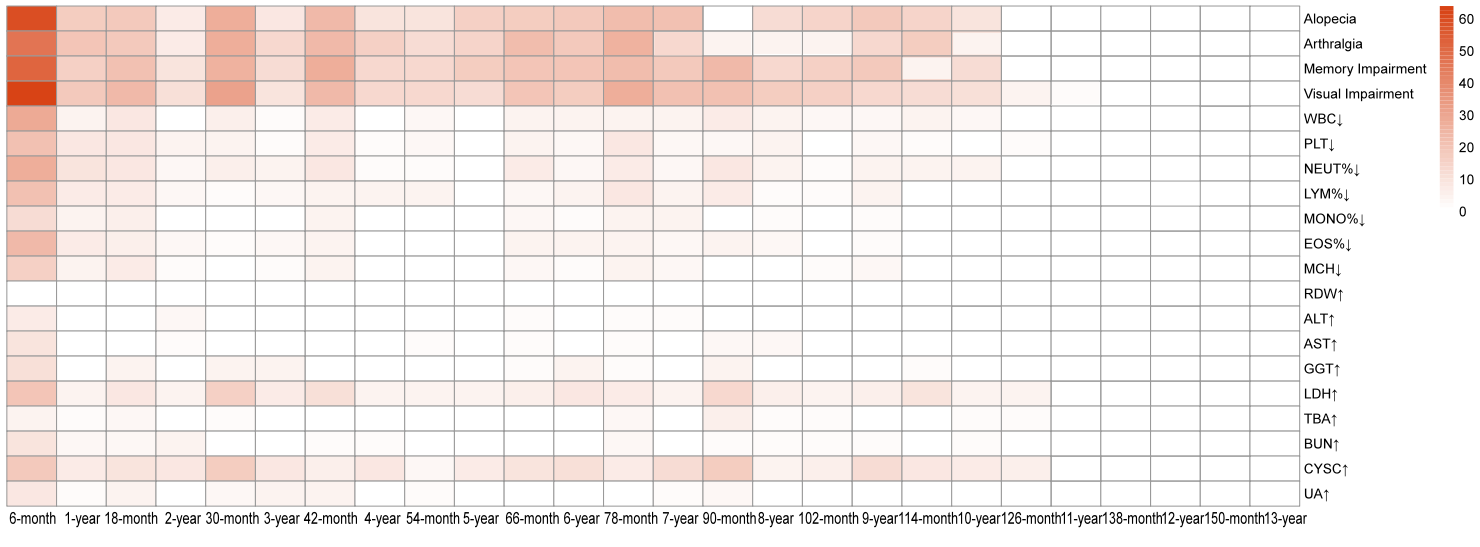

Supplement: S1 Fig — The heatmap uses color intensity to represent the number of individuals within each category, with darker colors indicating a higher number of individuals. The symbols ‘↓’ and ‘↑’ indicate laboratory values below and above the normal range, respectively. Abbreviations: BUN, blood urea nitrogen; CYSC, cystatin C; EOS%, eosinophil percentage; GGT, gamma-glutamyltransferase; LDH, lactate dehydrogenase; LYM%, lymphocyte percentage; MCH, mean corpuscular hemoglobin; MONO%, monocyte percentage; NEUT%, neutrophil percentage; PLT, platelet count; RDW, red cell distribution width; UA, uric acid; WBC, white blood cell count. (TIF) [file pntd.0013276.s015.tif]
