# Supplementary material for: Long-term clinical sequelae in severe fever with thrombocytopenia syndrome: A longitudinal cohort study
Source: PLoS Negl Trop Dis. 2025 Aug 12;19(8):e0013276. doi: 10.1371/journal.pntd.0013276 (PMC12360653; doi:10.1371/journal.pntd.0013276)
Supplement: S1 File — (DOCX) [file pntd.0013276.s016.docx]

**Questionnaire for assessing long-term clinical sequelae in survivors of severe fever with thrombocytopenia syndrome (SFTS)**

Purpose: To monitor clinical recovery progress and identify persistent sequelae requiring medical intervention in SFTS patients.

**Section 1: Demographic Information**

Follow-up Date (Day/Month/Year): __________________

1.Record: ____; Medical Record Number: __________________

2.Contact (WeChat/Phone): ______________________________

3.Age: ____

4.Gender:

☐ Male ☐ Female

5.Permanent address (Province/City/District): ________________________

**Section 2: Post-Recovery Symptom Assessment**

(Score 0-3 for each item: 0=None, 1=Mild, 2=Moderate, 3=Severe)

**Fatigue:**

6.Fatigue or mental exhaustion occurring after infection.

7.In the past few weeks, have you felt fatigued? Fatigue is characterized by physical and mental weakness or exhaustion; even after rest, you still feel tired.

8.In the past few weeks, did you feel interested in doing things, but found yourself lacking energy or in poor mental condition?

9.In the past few weeks, after resting or sleeping, did you feel that your physical strength was not as good as before or that you were physically exhausted?

10.In the past few weeks, after simple activities or work, did you feel that fatigue and mental exhaustion worsened?

**Memory Impairment:**

11.In the past few weeks, have you frequently forgotten recent events (e.g., conversations, appointments)?

12.In the past few weeks, have you struggled to recall names of familiar people or objects?

13.In the past few weeks, have you needed written reminders or alerts to complete daily tasks? 14.In the past few weeks, has memory loss caused misunderstandings in social interactions?

15.In the past few weeks, have you felt frustrated or anxious due to memory difficulties?

**Alopecia:**

16.Since infection, have you noticed significantly increased hair loss (e.g., clumps in the shower or on your pillow)?

17.In the past few weeks, has hair thinning become visibly noticeable to others?

18.In the past few weeks, have you avoided hairstyling (e.g., combing, tying hair) due to fear of hair loss?

19.In the past few weeks, has hair loss affected your self-confidence or social activities?

20.In the past few weeks, have you sought medical treatment or supplements for hair loss?

**Arthralgia:**

21.In the past few weeks, have you experienced joint stiffness or pain upon waking?

22.In the past few weeks, has joint pain worsened after physical activity (e.g., walking, climbing stairs)?

23.In the past few weeks, have you modified daily routines (e.g., using aids, avoiding movements) due to joint pain?

24.In the past few weeks, has joint pain disrupted your sleep quality?

25.In the past few weeks, have you used heat/cold therapy or massage for pain relief?

**Visual Impairment:**

26.In the past few weeks, have you experienced difficulty reading small print or screens?

27.In the past few weeks, have you had headaches or eye strain after visual tasks (e.g., reading, driving)?

28.In the past few weeks, have you needed to increase lighting for routine activities?

29.In the past few weeks, has visual impairment caused hesitation in unfamiliar environments (e.g., stairs, crowded places)?

30.In the past few weeks, have you consulted an eye specialist for new symptoms?

**Fever:**

31.In the past few weeks, have you experienced recurrent fever episodes (≥37.5°C) lasting over 24 hours?

32.In the past few weeks, during fevers, have you experienced muscle aches or loss of appetite? 33.In the past few weeks, have fevers forced you to cancel planned activities or work?

34.In the past few weeks, have you used antipyretics (e.g., paracetamol) more than twice weekly? 35.In the past few weeks, have you required emergency care for uncontrolled fever?

**Dizziness:**

36.In the past few weeks, have you felt dizzy when standing up quickly?

37.In the past few weeks, has dizziness occurred alongside rapid heartbeats or sweating?

38.In the past few weeks, have you restricted driving or exercise due to dizziness?

39.In the past few weeks, has dizziness made you dependent on others for daily tasks?

40.In the past few weeks, have you experienced vertigo (spinning sensation)?

**Headache:**

41.In the past few weeks, have headaches lasted longer than 4 hours without relief?

42.In the past few weeks, have headaches been triggered by specific factors (e.g., stress, light)?

43.In the past few weeks, have you missed work or school due to headaches?

44.In the past few weeks, have headaches caused nausea or vomiting?

45.In the past few weeks, have you avoided social events due to headache fears?

**Hearing Loss:**

46.In the past few weeks, have you misunderstood conversations in noisy environments?

47.In the past few weeks, have you increased TV/device volume beyond others’ comfort?

48.In the past few weeks, has hearing loss caused embarrassment in group settings?

49.In the past few weeks, have you experienced ear pain or pressure alongside hearing issues?

50.In the past few weeks, have you considered hearing aids or medical evaluation?

**Myalgia:**

51.In the past few weeks, have you experienced muscle cramps or spasms?

52.In the past few weeks, has muscle pain localized to specific areas (e.g., neck, calves)?

53.In the past few weeks, have you avoided stretching or exercise due to muscle pain?

54.In the past few weeks, has myalgia interfered with sitting or standing for long periods?

55.In the past few weeks, have you used topical analgesics (e.g., creams, patches)?

**Section 3: Medical & Exposure History**

Post-Discharge Tick Exposure & Recurrence

Have you been bitten by ticks since hospital discharge?
☐ Yes ☐ No

Have you experienced recurrent acute fever or SFTS-like symptoms since discharge?
☐ Yes (Date: ___________) ☐ No

Do you raise livestock/pets at home? (Check all that apply)
☐ Cattle ☐ Sheep ☐ Pigs ☐ Dogs ☐ Chickens ☐ Geese ☐ None

Has any family member been diagnosed with SFTS (Severe Fever with Thrombocytopenia Syndrome)?
☐ Yes ☐ No
If Yes:

Relationship to you: ___________________

Name: ___________________

Pre-existing Chronic Conditions

Do you have any of the following chronic diseases?

Diabetes: ☐ Yes ☐ No

Hypertension: ☐ Yes ☐ No

Other chronic diseases: ___________________

For Hypertension Patients:

Medication name(s): ___________________

Duration of use: _______ years

For Diabetes Patients:

Medication name(s): ___________________

Duration of use: _______ years
